# In pursuit of saccade awareness: Limited volitional control and minimal conscious access to catch-up saccades during smooth pursuit eye movements

**DOI:** 10.1167/jov.26.6.10

**Published:** 2026-06-25

**Authors:** Jan-Nikolas Klanke, Sven Ohl, Almila Naz Esen, Martin Rolfs

**Affiliations:** 1 Berlin School of Mind and Brain, Humboldt-Universität zu Berlin, Berlin, Germany; 2 Department of Psychology, Humboldt-Universität zu Berlin, Berlin, Germany; 3 Department of Applied Human Sciences, Hochschule Magdeburg-Stendal, Magdeburg, Germany; 4 Department of Psychology, University of Potsdam, Potsdam, Germany; 5 Bernstein Center for Computational Neuroscience Berlin, Humboldt-Universität zu Berlin, Berlin, Germany

**Keywords:** catch-up saccade, pursuit eye movement, motor control, awareness

## Abstract

Observers use smooth pursuit to track moving objects—like koi carp gliding through a pond. When positional errors accumulate, rapid catch-up saccades correct for them. Despite their abruptness, these saccades usually go unnoticed, creating the seamless experience of smooth tracking. We conducted three experiments to examine awareness and control of catch-up saccades (Experiment 1), the effect of training (Experiment 2), and of movement intention (Experiment 3). All experiments followed a similar protocol. On each trial, a target moved horizontally at one of three constant speeds (3–12 dva/s). Two horizontal stimulus bands with vertically oriented gratings appeared above and below the trajectory. These bands were rendered invisible during pursuit by rapid phase shifts (>60 Hz), but became visible when briefly stabilized on the retina—either by a catch-up saccade or its replayed retinal consequence—providing immediate, saccade-contingent visual feedback. Observers reported whether they had seen the stimulus bands (visual sensitivity) and whether they were aware of making a catch-up saccade (saccade sensitivity). Visual sensitivity was consistently higher in trials with a catch-up saccade, confirming that these movements reduce retinal motion and enhance visibility. Higher target speeds increased saccade rate, but observers struggled to control them volitionally: Visual feedback and training had no effect on the ability to control catch-up saccades. Only suppression-instructions yielded a small reduction. Saccade sensitivity was near zero, even in trials with saccade-contingent feedback. Neither training nor intention improved awareness. Together, our data suggest a limited ability to control and a low level of awareness of catch-up saccades during pursuit.

## Introduction

Picture a koi pond, where vibrant carp glide effortlessly beneath the surface. One koi, with a particularly striking pattern, catches your attention, and you begin to track its path through the shifting background of other colorful fish. To focus on the koi, and follow its motion through the water, your eyes engage in a behavior called “pursuit”—a slow, smooth rotation of the eyes that fixes your center of gaze on a moving target without loss in visual sensitivity (Schütz, Braun, Kerzel, & Gegenfurtner, 2008; Schütz, Braun, & Gegenfurtner, 2009).^1^ Consequently, pursuit perfectly explains your stable, and detailed (high-resolution) impression of the fish on its trajectory through the pond. However, initiating and maintaining pursuit is limited by reaction time, and target speeds are neither reached instantly nor sustained perfectly (Goettker & Gegenfurtner, 2021), leading to deviations between the intended and actual gaze positions. To correct for these position errors, observers frequently initiate catch-up saccades: rapid eye movements that realign the center of gaze with the moving target (De Brouwer, Yuksel, Blohm, Missal, & Lefèvre, 2002). Although catch-up saccades are necessary for successful tracking, they clash with how we experience pursuit: when tracking the koi in its pond, our impression is not of a jerky or unstable fish, but of one that remains fixed at the center of gaze while gliding smoothly through the water. Likewise, we feel as though our eyes move continuously and smoothly with the fish, rather than being frequently interrupted by abrupt, ballistic shifts in gaze position. In this study, we examine the discrepancy between the objective presence of catch-up saccades in gaze behavior and the subjective experience of smooth tracking. Specifically, we set out to address two key questions: First, we examine whether observers can voluntarily control catch-up saccades, that is, suppress or generate these movements at will. Second, we investigate their awareness of catch-up saccades during pursuit, evaluating observers’ ability to detect when a movement has been executed.

Effective motor control is fundamental to everyday behavior, and this is particularly evident for eye movements, which supply the visual information that guides many other actions. To support this role, observers must be able to direct their eyes deliberately and flexibly. For “regular” saccades, such volitional control is well established: when emphasized by task demands, saccades can redirect the gaze to new targets with remarkable spatial (Kowler & Blaser, 1995) and temporal (Kinder, Rolfs, & Kliegl, 2008; Wong & Shelhamer, 2012; Zingale & Kowler, 1987) precision. This capacity suggests that the ability to act requires a rudimentary form of (prospective) action awareness—an awareness that enables intentionally shaped movement behavior. Support for this notion comes from research demonstrating that awareness is far less reliable for reflexive movements: Reflexive oculomotor responses, such as those elicited in an anti-saccade task (Mokler & Fischer, 1999) or during oculomotor capture (Belopolsky, Kramer, & Theeuwes, 2008; Theeuwes, Kramer, Hahn, & Irwin, 1998), can escape awareness approximately half the time. These insights, however, come predominantly from paradigms involving discrete movements. Catch-up saccades are generated during pursuit and are driven by position errors and visual motion—much like pursuit itself (Goettker & Gegenfurtner, 2021; Krauzlis, 2004; Rashbass, 1961). They are thus inherently embedded within the continuous dynamics of smooth eye movements and constitute a distinct sensorimotor regime from discrete saccades. This allows us to examine volitional control and awareness in the context of continuous, ongoing movements. How awareness emerges in this context remains unclear. The roles of visual feedback, efference copy, and proprioception—and the ways they interact—are equally uncertain, and their contributions to shaping awareness are not yet understood.

Despite evidence that catch-up saccades are an automatic response to position or velocity errors during pursuit (De Brouwer et al., 2002; Nachmani, Coutinho, Khan, Lefèvre, & Blohm, 2020), it remains unclear whether they are entirely beyond voluntary control or if some level of control can still be exerted. Our first research objective was, therefore, to test whether observers can suppress (or at least postpone) catch-up saccades, or if these movements are invariantly triggered when the conditions for their generation are met. Catch-up saccades are equally interesting when it comes to awareness: They are mostly reflexive eye movements that are frequent, small, and fast, and, hence, have the potential to escape awareness (much like spontaneous microsaccades, cf., Klanke, Ohl, & Rolfs, 2025b). As eye movements that are accompanied by visual transients as well as clear markers of success (i.e., the shift in the tracked object's retinal position from peripheral to foveal), however, catch-up saccades might also be generated with a heightened degree of conscious oversight. This ambivalence makes them ideal for exploring our second research objective: understanding observers’ awareness of their catch-up saccades and the factors that modulate it. Closely tied to the question of awareness of catch-up saccades is the role of visual perception. When tracking a target, we have the impression of continuous foveation, without any noticeable disruptions. This perceptual continuity suggests that visual mechanisms contribute to masking the abrupt retinal shifts caused by catch-up saccades, preserving the illusion of smooth and uninterrupted pursuit. Our third research objective was therefore to examine how these perceptual processes interact with awareness of catch-up saccades.

**Figure 1.**
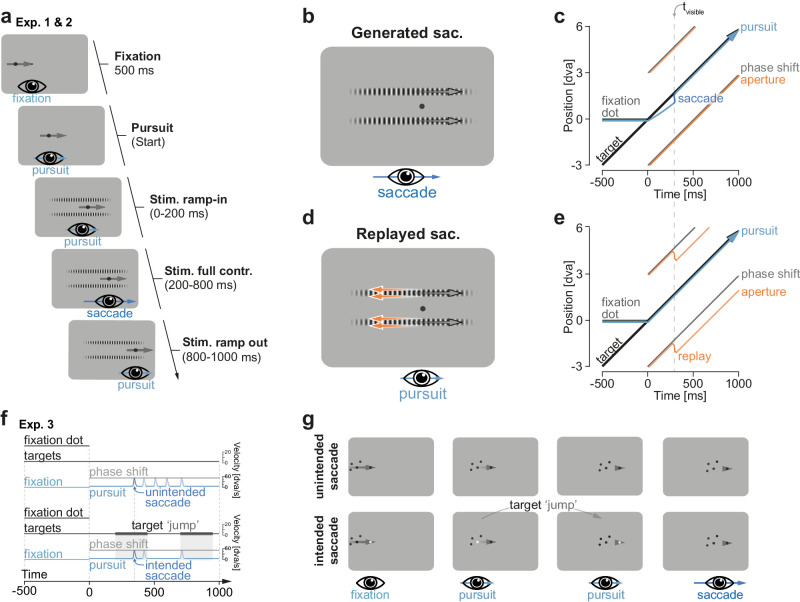
Experimental protocol and stimulus design. (**a**) Procedure of Experiments 1 and 2. Participants had to fixate for 500 ms while the movement target moved towards the fixation location at a constant speed of either 3, 6, 9, or 12 dva/s. They had to start pursuing, once the target fully occluded the fixation dot. Pursuit and stimulus interval lasted for 1000 ms, with stimulus bands increasing to 50% contrast during the first 200 ms—and decreasing to 0% in the 200 ms of each trial. (**b**) Stimulus display for generated catch-up saccades. Gray arrows indicate the direction of the phase shift; blue arrow indicates the direction of a catch-up saccade that leads to a retinal stabilization of the stimulus. (**c**) Spatiotemporal configuration of fixation dot, movement target, eye position, and stimulus (aperture and phase) during a trial (schematic, for actual gaze traces, velocity, and acceleration data see Figure 3). The time of the saccade (t_visible_) marks the moment when the generated saccade stabilizes the phase shift on the retina. (**d**) Stimulus display for replayed catch-up saccades. Gray arrows indicate the direction of the phase shift (as in panel b), whereas orange arrows indicate the direction of an aperture shift that replicates the retinal consequences of a saccade, resulting in retinal stabilization of the stimulus similar to the saccade shown in panel b. (**e**) Spatiotemporal configuration of fixation dot, movement target, eye position, and stimulus (aperture and phase) during a replay trial. The time of stimulus visibility (t_visible_) is aligned with an aperture motion that replicates the retinal consequences of a saccade. (**f**) Procedure during unintended and intended catch-up saccade conditions in Experiment 3. In the unintended saccade condition, the procedure closely matched that of the first two experiments (e.g., without a target jump), with all saccades occurring unintentionally. In the intended condition, gray bands indicate the timing of the early or late target jumps (e.g., the brief recoloring of the current and future pursuit targets; see panel g for details). (**g**) Instruction conditions in Experiment 3. Unintended saccade condition (upper row): Participants were instructed to fixate on a black dot (panel 1) until one dot from the moving cloud crossed the fixation point (panel 2). They then tracked the black dot as it moved (panel 3), allowing spontaneous catch-up saccades to occur without explicit instruction (panel 4). Intended saccade condition (lower row): Participants were instructed to fixate on a white dot (panel 1) until it was crossed by a moving dot from the cloud, which turned white upon fully occluding the fixation dot (panel 2). They then tracked the moving white dot until it turned black, and another dot flashed white for 50 ms (panel 3), serving as the go-signal to make a catch-up saccade. Subsequent saccades were thus labeled intended.

To systematically investigate voluntary control and awareness of catch-up saccades, we conducted three experiments in which participants tracked a continuously moving target (Figures 1a, 1c). Across experiments, catch-up saccade rate was taken as an indicator of volitional saccade control, providing a measure of participants’ ability to modulate their eye movements. Trial-by-trial reports of perceived catch-up saccade generation provided a measure of eye movement awareness. To additionally test if the perceptual consequences of a catch-up saccade facilitated its detection, we controlled the visual information available during these movements by adding a high-speed stimulus that was generally invisible during pursuit (Figures 1b, 1c). A catch-up saccade, could briefly stabilize it on the retina, rendering it visible as immediate saccade-contingent feedback. To ensure that the visual event alone did not fully determine saccade detection, we included a condition that replayed the retinal motion caused by a saccade without requiring an actual eye movement (Figures 1d, 1e), as well as a no-stimulus condition. From participants’ reports of stimulus perception, we calculated visual sensitivity to determine the stimulus provided informative, saccade-contingent feedback. To isolate different contributing factors, each experiment additionally introduced a specific variation. Experiment 1 established the paradigm and assessed baseline sensitivity, both visual and saccadic. Experiment 2 tested whether participants could train voluntary control or awareness of catch-up saccades. Experiment 3 manipulated movement intention: participants were either instructed to pursue the target without generating a catch-up saccade (Figures 1f, 1g, upper row), or to generate a specific saccade (Figures 1f, 1g, lower row).

## Methods

### Participants

#### Experiment 1

In Experiment 1, a total of eight participants were recruited by means of the “Psychologischer Experimental-Server Adlershof” of the Humboldt-Universität zu Berlin and from members of the laboratory. Participants (four female, zero diverse) had a mean age of 25 years (*SD =* 3.9, *min* = 21, *max* = 33). Of our participants, seven were right-handed and one was left-handed. Similarly, seven were right-eye dominant, and one participant was left-eye dominant. All eight participants had normal or corrected-to-normal vision. Participants were paid on completion of the last session. The compensation was based on an hourly rate of €10/hour. Alternatively, psychology students could choose to obtain participation credit (1 credit per 15 minutes of participation) required for the successful completion of their bachelors’ program. The study was approved by the ethics committee (Ethikkommission) of the Institut für Psychologie at the Humboldt-Universität zu Berlin and conducted in agreement with the Declaration of Helsinki (World Medical Association Declaration of Helsinki, 2013) and the General Data Protection Regulation of the EU. All participants provided informed consent in writing before the start of the first session.

#### Variations in Experiment 2

Experiment 2 followed the same procedure with a total of ten participants recruited via the same channels. Participants (nine female, 0 diverse) had a mean age of 25.5 years old (*SD =* 3.3, *min* = 21, *max* = 30). Of these participants, nine were right-handed and one was left-handed. Similarly, nine participants were right-eye dominant, one participant was left-eye dominant. All ten participants had normal or corrected-to-normal vision.

#### Variations in Experiment 3

Experiment 3 followed the same procedure as well. We recruited a total of ten participants again, (seven female, one diverse) had a mean age of 22.7 years old (*SD* = 2.1, *min* = 20, *max* = 25). All ten were right-handed and six were right-eye dominant. All ten participants had normal or corrected-to-normal vision.

#### Exclusion of participants

For Experiments 1, 2, and 3, we pre-registered an exclusion criterion that ensured that participants would not participate if they showed the inability to execute stable fixation or correct eye movements: The inability to complete at least four blocks during the first experimental session due to fixation failures led to immediate exclusion from the experiment in all experiments. Across experiments, data collection continued until the full preregistered sample size was reached: eight participants for Experiment 1, and ten for Experiments 2 and 3.

##### Experiment 1

In Experiment 1, no participants were excluded from data collection; however, one participant chose to discontinue their participation after completing the first session for personal reasons. As a result, this participant's data was excluded from all analyses.

##### Variations in Experiment 2

In Experiment 2, one participant was excluded due to an eye tracker malfunction that occurred during the fifth block of the first session, resulting in no data being saved for the entire session. To minimize the impact of missing data, we discontinued the experiment for this participant. Additionally, two other participants withdrew after partially completing the first session for personal reasons, and their data was likewise excluded from all analyses.

##### Variations in Experiment 3

In Experiment 3, no participants were excluded, but one chose to discontinue after completing two of the four sessions for personal reasons. This participant's data was excluded from all analyses.

### Materials and procedure

#### Apparatus

In Experiments 1, 2, and 3, participants were seated in a dark room in front of a screen at a distance of 340 cm and their head stabilized using a chin rest. We projected visual stimuli on a 141.0 × 250.2 cm video-projection screen (Stewart Silver 5D Deluxe; Stewart Filmscreen, Torrance, CA, USA) using a PROPixx DLP (960 × 540 pixels; VPixx Technologies Inc., Saint Bruno, Canada) with a refresh rate of 1440 Hz. We recorded participants’ eye positions of both eyes with a head-mounted eye tracker at a sampling rate of 500 Hz (EyeLink 2 Head Mount; SR Research, Ottawa, Canada). The experiments were controlled on a workstation running the Debian 8 operating system, using Matlab (MathWorks, Natick, MA, USA), the Psychophysics Toolbox 3 (Brainard, 1997; Kleiner et al., 2007; Pelli, 1997) and the EyeLink Toolbox (Cornelissen, Peters, & Palmer, 2002).

#### Eye movement task

##### Experiment 1

To examine control and awareness of catch-up saccades during pursuit, we deployed a paradigm that provided consistent retinal motion across trials while preserving natural catch-up saccades, making it more suitable for studying volitional control and awareness than a classic step-ramp Rashbass stimulus. In our adaptation, participants tracked a black target (0.35 dva diameter) moving in a straight horizontal line across the screen midline; target velocities were 6, 9, or 12 dva/s in Experiment 1 corresponding to movement amplitudes of 6, 9, or 12 dva. To facilitate pursuit initiation without early saccades, each trial began with a fixation interval during which participants maintained gaze on a central fixation dot while the moving target—initially offset by 3, 4.5, or 6 dva—was already in motion toward the fixation point. This setup ensured that the pursuit target crossed the fixation location at the moment pursuit was to begin, enabling smooth tracking without the need for a corrective catch-up saccade. Participants were instructed to maintain fixation until the target reached the fixation location, after which they were to pursue the target smoothly. Target motion was continuous throughout the trial, with no pauses or halts. Target velocity was blocked, and participants were informed before each block whether target speed would be low, medium, or high. Requiring an initial 500 ms fixation period while the target was already in motion allowed the paradigm to balance stable gaze before pursuit onset with consistent retinal slip across trials. It also helped avoid the variability in early retinal motion typical of classic step-ramp procedures. Additionally, this stimulus preserved natural catch-up behavior and was thus especially suitable for our study, which focused on volitional control and awareness of catch-up saccades (for an analysis illustrating saccade behavior across experiments, see Supplementary Material S5: Smoothed saccade rates)*.*

##### Variations in Experiment 2

In Experiment 2, the eye movement task was identical to the first experiment, with the only difference being that target speeds were lowered to 3, 6, and 9 dva/s (with movement amplitudes reduced accordingly to 3, 6, and 9 dva). Consequently, the initial dot offset was halved to 1.5, 3, or 4.5 dva. All other parameters remained as in Experiment 1.

##### Variations in Experiment 3

In Experiment 3, we extended this approach by using more visually complex pursuit targets to probe the influence of intentional control on eye movement awareness (see next paragraph for details). Instead of a single moving dot, the pursuit target consisted of a cloud of 4 to 8 black dots (each 0.22 dva in diameter), with dot positions sampled from a 2D Gaussian distribution (x: *M* = 0, *SD* = 1 dva; y: *M* = 0, *SD* = 0.4 dva), resulting in a horizontally elongated shape. Targets moved horizontally across the screen midline—either left to right or right to left—at constant speeds of 3, 6, or 9 dva/s (same as in Experiment 2), covering distances of 3, 6, or 9 dva, respectively.

To investigate the role of intention in saccade awareness during pursuit, each trial in Experiment 3 featured one of two eye movement instructions. In unintended saccade trials, participants were instructed to pursue the target as smoothly as possible; here, both the fixation point and all target dots were black, and any catch-up saccades were reflexive. In contrast, intended saccade trials required participants to deliberately generate a saccade during pursuit. These trials began with a white fixation dot that disappeared as soon as it was occluded by one of the moving target dots, which in turn changed to white to indicate the dot to be pursued. This dot remained white for 200–450 ms (early jump) or 700–950 ms (late jump) relative to pursuit onset, after which a second target dot turned white for 50 ms to signal the saccade target. Stable gaze during this interval (the initial 500 ms fixation period; see *Eye movement task,* Experiment 1) was particularly important in Experiment 3, as movement instructions were presented at this time. All dots then returned to black, indicating that the participant should execute the instructed saccade. Jump targets were offset by 0.5, 1, or 1.5 dva horizontally (left or right of the pursuit target) and included a vertical offset of ±5° to introduce oblique saccades and reduce trial predictability. The early and late jump conditions were designed to manipulate participants’ ability to comply with the saccade instruction, with early jumps facilitating and late jumps potentially hindering timely execution.

#### The stimulus

##### Experiment 1

**Figure 2.**
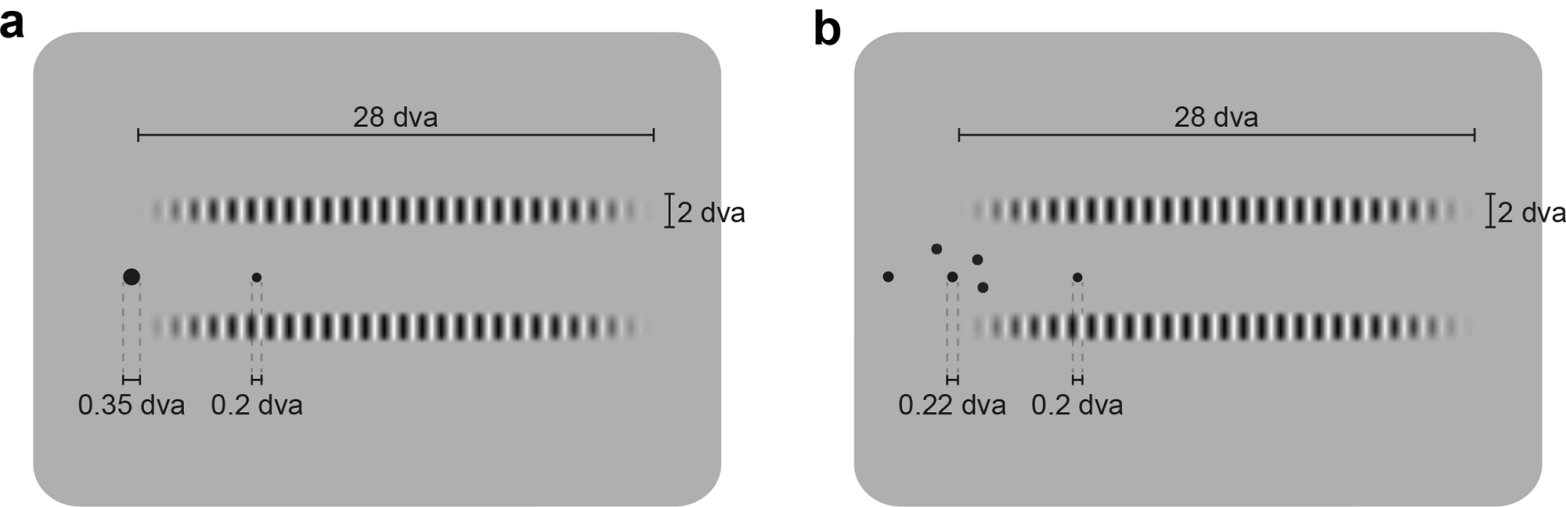
Illustration of the stimulus bands and pursuit targets. (**a**) Stimulus bands and pursuit targets as presented in Experiments 1 and 2. (**b**) Stimulus bands and pursuit targets as presented in Experiment 3.

The stimulus was formed by two vertically oriented sinusoidal gratings with spatial frequency of 5 cpd, combined with identical cosine-tapered masks. The combined gratings and masks appeared like two striped horizontal bars that smoothly blended in with the gray background (see Figures 1b and 1d). Both stimuli had a length of 28 dva and height of 2 dva (see Figure 2^3^a). The size of the tapered sections is always 1. Masks were created by generating separate cosine tapered windows for the height and the width of each stimulus that are then combined by multiplication (again, separately per each stimulus).

**Figure 3.**
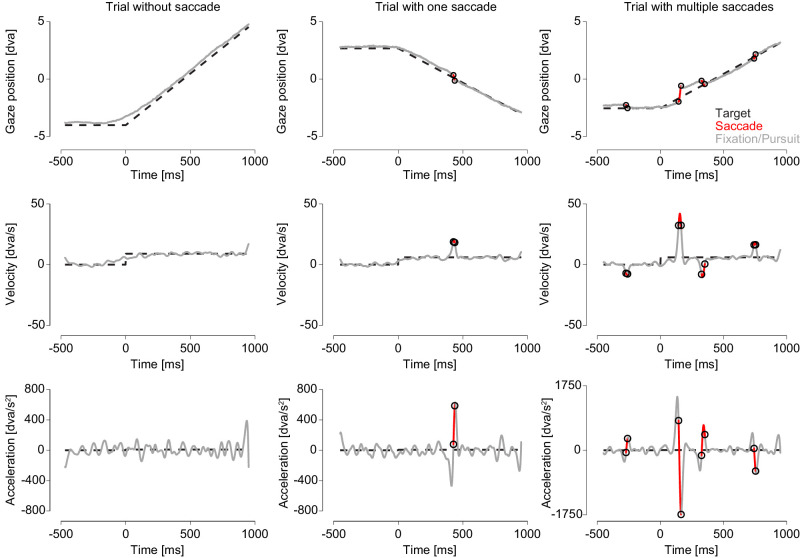
Detection of zero (left), one (middle), or multiple saccades (right column) by the co-registered detection approach. Data show raw position (top), velocity derived from smoothed positions (middle), and acceleration computed from these velocities before final smoothing (bottom) of three randomly selected trials of one participant. Saccade onsets and offsets (circular markers) were detected based on the raw position data (top row). The velocity and acceleration traces are displayed after low-pass filtering (30 Hz), which broadens their peaks. As a result, the marked saccade intervals do not span the full width of the filtered velocity/acceleration traces. Note that the y-axis of the rightmost acceleration plot differs from the others due to the large magnitude of the negative deflection.

To examine if a visual consequence affected awareness of the underlying eye movement, our stimulus was designed to be invisible during pursuit, but visible when briefly stabilized on the retina by a saccade. To achieve invisibility, we added a high-velocity phase shift to the grating, creating a temporal frequency above 60 Hz (cf., Castet & Masson, 2000). In Experiment 1, phase shift was based on the peak velocity of a saccade with an amplitude of 2 dva (i.e., 100.65 dva/s according to the formula and values reported by Collewijn, Erkelens, & Steinman, 1988). We added the speed of the pursuit target to the phase shift velocity to ensure invisibility in the absence of a saccade, leading to phase shift velocities of 106.65, 109.65, and 112.65 dva/s respectively. The direction of the phase shift—solely determined by the stimulus orientation—can be either rightward (stimulus orientation of 0° from vertical) or leftward (stimulus orientation of 180° from vertical) to match the direction of potential catch-up saccades (the smooth pursuit target will be moving purely horizontally). In Experiment 1, phase shifts were always oriented in the same direction. Stimulus presentation started at a contrast of 0 and was ramped up to maximum contrast of 50% within 200 ms. Conversely, in the last 200 ms of presentation, the stimulus contrast slowly decreased, and the stimulus faded out. The stimulus was thusly modulated to avoid sudden onsets and offsets that might have led to transient changes in stimulus visibility. Because generated catch-up saccades rendered it visible, this stimulus condition was called *generated saccade* condition throughout Experiments 1, 2, and 3.

Presented in this way, the stimulus should become visible only during catch-up saccades, which transiently reduce the retinal velocity of the grating. This momentary stabilization can raise the grating above the detection threshold, much like how saccades (or microsaccades) have been shown to reveal otherwise invisible high temporal frequency patterns (Deubel, Elsner, & Hauske, 1987; Deubel & Elsner, 1986; Kelly, 1990). In our paradigm, stimulus visibility was therefore a graded phenomenon that depended on retinal velocity: high retinal velocity kept the grating invisible, partial stabilization produced a faint or blurry impression, and low retinal velocity—achieved when saccade direction matched the phase shift and peak velocity matched the stimulus speed—produced a “short, distinct flash” (Deubel et al., 1987). Saccades in the opposite direction increased retinal velocity, maintaining invisibility of our stimulus. Because the pursuit target shifted horizontally, horizontal saccades modulated visibility most strongly: catch-up saccades the same direction rendered it highly visible, whereas saccades in opposite direction suppressed its perception. Consequently, in Experiment 1, where both gratings shifted in the same direction, participants could not see the stimulus if saccade and phase-shift directions mismatched.

##### Variations in Experiment 2

Because of a poor stimulus visibility in the first experiment and the lower pursuit target speeds, we decided to use a lower phase shift velocity in Experiment 2. Here, the phase shift velocity was based on a saccade with an amplitude of 1 dva, resulting in a base velocity of 53.50 dva/s. After correcting for the speed of the pursuit target, the phase shift velocity was set to be 56.50, 59.50, or 62.50 dva/s.

In addition, to increase stimulus visibility, phase shifts were oriented in opposite directions in Experiment 2 (whereas gratings had shifted in the same direction in each trial in Experiment 1). With gratings shifting in opposite directions, catch-up saccades in both horizontal directions enhanced the visibility of one of the gratings; either the one presented above or the one below the pursuit trajectory.

##### Variations in Experiment 3

In Experiment 3, the phase shift parameters were identical to Experiment 2, with the only difference being the replacement of the pursuit target (see *Task,* Variations in Experiment 3; Figure 2b).

#### Replay condition

##### Experiment 1

We compared stimulus visibility for generated saccades with two other conditions: In one condition, we used the same stimulus (as described in the previous paragraph) in terms of its spatial frequency, size, the extent of its tapered section, its orientation, contrast modulation, phase shift, as well as phase onset, placement, and presentation duration. We added a rapid change in the onscreen locations of the stimulus apertures to replicate the retinal consequence of a typical eye movement for the observer. If the aperture movement was in the opposite direction of the stimulus' phase shift, the image of the stimulus appeared to slow down on the retina, resulting in retinal effects very similar to those of an actual catch-up saccade. The aperture motion was generated based on a fixed set of parameters and simulated a catch-up saccade of 2 dva and a peak velocity of 100.65 dva/s in all three sessions of Experiment 1.

Finally, in the *no-stimulus condition* trials, the stimulus will be presented at 0% contrast whereas everything else will be identical to trials with generated and replayed saccades. Twenty percent of all trials were no-stimulus condition trials while the remaining 80% of trials will be with stimulus. Stimulus trials were split evenly between generated (40%) and replayed saccades (40%).

##### Variations in Experiment 2

In the first session of Experiment 2, aperture motion was based on the parameters of a saccade with an amplitude of 1 dva and a peak velocity of 53.5 dva (to match the lower phase shift velocity of the stimuli; see *The stimulus*, Variations in Experiment 2). In the later sessions, we adapted these parameters to the eye movement data of each participant to better match the stimulus visibility for generated catch-up saccades: we fit gamma functions to the distribution of saccade amplitudes measured for the three different target speed conditions (i.e., 3, 6, and 9 dva/s). We additionally fitted main sequence functions to the saccade amplitude and peak velocity data to determine the optimal peak velocity at any given amplitude for each observer (the parameters and formula for calculating the velocity profile of these simulated catch-up saccades were based on Collewijn et al., 1988). During the experiment, we sampled individual saccade amplitudes from observer-specific gamma distributions and determined corresponding peak velocities based on the main sequence fits. These parameters were then used to simulate biologically plausible, observer-specific catch-up saccade profiles, which were replayed via aperture movements. The direction of these simulated saccades was constrained to fall within ±2° of the horizontal axis (i.e., 0° or 180°). We called this condition *replay condition.*

To increase stimulus visibility and make it a more reliable predictor of saccade generation, we increased of number of generated saccade trials to 60% in Experiment 2. The remaining trials were split between the replayed saccade (20%) and no-stimulus condition (20%).

##### Variations in Experiment 3

All parameters were identical to Experiment 2, except that we returned to a more even split between conditions and presented the stimulus 37.5% of trials in the generated saccade condition, 37.5% in the replayed saccade condition, and in 25% of trials in the no-stimulus condition.

### Procedure

#### Experiment 1

##### Fixation-check interval

Before the start of each trial, a target-shaped fixation dot appeared before an otherwise gray background. The fixation point (inner part) had a diameter of 0.2 dva while the outer ring had a diameter of 0.6 dva. Before the onset of each trial, a fixation control routine was run that required the gaze position of the observer to be inside a circular region (3 dva in diameter) centered on the fixation dot. The trial started only when the fixation control was successful for at least 100 ms.

##### Fixation interval

The start of the fixation interval was marked by the disappearance of the outer ring of the fixation point. Participants were instructed to look at the fixation dot and not move their eyes for the entire duration of this phase of the trial (500 ms). At the same time, a pursuit target appeared at either 3, 4.5, or 6 dva relative to the fixation dot, positioned towards the outer screen edge (opposite to the motion direction). It moved with constant speed of 6, 9, or 12 dva/s toward the fixation dot. The target was a black dot with a diameter of 0.35 dva. Participants were instructed to keep fixating until the target reached the fixation dot.

##### Pursuit and stimulus presentation interval

The pursuit and stimulus presentation interval began once the pursuit target fully occluded the fixation dot (i.e., the observer's gaze position). Participants were instructed to start pursuing the target with their eyes when this occurred. In active or replay condition trials, two stimuli were presented 3 dva above and below the screen's horizontal midline. The bands had a length of 28 dva and a height of 2 dva. The pursuit and stimulus presentation interval lasted for 1000 ms in total. Between this and the response interval, there was a short delay of 50 ms during which nothing was presented onscreen.

##### Response interval

In the response interval, participants were always presented with two simple yes-no questions Firstly, we asked participants if they perceived the stimulus in the previous trial. We presented the question “Did you perceive a STIMULUS FLASH?”, together with the two response options “Yes!” and “No!”. In a second step, participants reported if they generated a catch-up saccade. We displayed the question “Do you think you generated a CATCH-UP SACCADE?” and the same response options “Yes!” and “No!” Both questions could be answered by pressing the arrow key corresponding to the direction of the chosen response option (e.g., the right arrow key for a selected of the right-ward response prompt).

Participants’ responses to these first two questions determined the presentation of the final stage of the response phase—the link between eye movement and stimulus visibility: If participants reported that they perceived a stimulus flash and that they think they generated an eye movement, they were asked: “How sure are you that the stimulus WAS caused by a catch-up saccade?” If they reported that they did not perceive the stimulus, but thought they generated an eye movement, we asked “How sure are you that the stimulus flash was NOT caused by a catch-up saccade?” To answer, participants had to choose one of four options displayed on a continuous scale: “not sure,” “rather not sure,” “rather sure,” and “very sure.” Participants chose their response by adjusting the position of a response prompt via the arrow keys and submitted their response by pressing the space bar.

#### Variations in Experiments 2

We used the same procedure in Experiment 2 as in the first experiment with only minor variations: Target speeds were lowered to 3, 6, and 9 dva/s due to the high number of catch-up saccades in all target speed conditions of Experiment 1. Initial target positions were, therefore, adjusted to 1.5, 3, and 4.5 dva relative to the fixation dot. Because, unlike in the first experiment, the phase shift of the two stimuli bands was always in opposite directions in Experiment 2, we added a simple localization task to the response interval: If participants reported that they perceived the stimulus in response to the first question, we asked if they perceived the one above or below the midline of the screen (i.e., above or below the pursuit target trajectory). Participants could respond by pressing the up-arrow to indicate that they saw the stimulus above the screens’ midline, or the down-arrow if they perceived the one in the lower half of the screen. To keep the response interval concise, we omitted the final phase of the first experiment in Experiment 2, ending after asking if the participants thought they had generated a catch-up saccade (see Table 1 for an overview of the differences between experiments).

**Table 1.** Stimulus parameters and proportion of stimulus conditions used in Experiments 1–3.

	Experiment 1	Experiment 2	Experiment 3
Pursuit target velocity	6, 9, 12 dva/s	3, 6, 9 dva/s	3, 6, 9 dva/s
Stimulus phase shift velocity	106.65, 109.65, 112.65 dva/s	56.50, 59.50, 62.50 dva/s	56.50, 59.50, 62.50 dva/s
Stimulus phase shift directions	Parallel	Antiparallel	Antiparallel
Replayed saccades			
First session	Amplitude: 2 dva;	Amplitude: 1 dva	Amplitude: 1 dva
	Peak vel.: 100.65 dva/s	Peak vel.: 53.50 dva/s	Peak vel.: 53.50 dva/s
Later sessions	Amp. 2 dva;	Individualized	Individualized
	Peak vel. 100.65 dva/s		
Stimulus conditions			
No-stimulus	20%	20%	25%
Generated	40%	60%	37.5%
Replayed	40%	20%	37.5%

To examine if participants could be trained to suppress their catch-up saccades (and awareness thereof), participants were instructed in the beginning of the experiment to try and pursue as smoothly as possible. They were additionally informed that perceiving the stimulus likely indicated that a catch-up saccade was (accidentally) generated. Abbreviated instructions were presented before each session as a short reminder.

#### Variations in Experiment 3

The procedure of Experiment 3 was largely identical to that of Experiment 2. We used the same target speeds, stimulus phase shift directions, and response schema (see Table 1 for an overview of the differences between experiments). All other aspects of the task were kept constant, with the exception of two key changes: (1) the pursuit target was no longer a single dot but a dot cloud composed of four to eight smaller dots with randomly jittered x- and y-positions to create a horizontally elongated shape, and (2) we introduced trial-wise instruction cues to manipulate the intention behind catch-up saccades. Specifically, participants were asked to either pursue the target as smoothly as possible (unintended saccade condition) or to make an instructed saccade to a briefly highlighted target dot at a defined moment during pursuit (intended saccade condition). These saccade cues appeared either early (200–450 ms after pursuit onset) or late (700–950 ms), allowing us to examine how timing affected compliance with the instruction. The saccade targets were offset horizontally (±0.5, 1, or 1.5 dva) and vertically (±5°) relative to the initial pursuit dot to promote both forward/backward and oblique saccades. This manipulation enabled us to investigate participants’ awareness of both intended and unintended catch-up saccades.

### Online monitoring of eye positions

During Experiments 1, 2, and 3 participants’ eye positions were tracked. Eye and screen coordinates were aligned by conducting standard nine-point calibration and validation procedures before the first trial of each session and whenever necessary. Blinks and deviations in gaze position (>1.5 dva from fixation during the fixation interval, >9 dva from the target dot during the pursuit interval) were likewise monitored in all experiments and led to an abortion of the trial. Aborted trials were repeated at the end of each block in randomized order.

### Saccade detection

Binocular catch-up saccades were detected in Experiments 1, 2, and 3 using a combination of an acceleration-based threshold and the algorithm described by Engbert and Mergenthaler (2006). For the acceleration-based approach, we sequentially applied low-pass Butterworth filters to the position, velocity, and acceleration data for each component of the binocular eye-tracking signal, using a cutoff frequency of 15 Hz for position and 30 Hz for both velocity and acceleration (c.f., Fooken & Spering, 2020; Harris et al., 2023). If acceleration exceeded a detection threshold of at least 300 dva/s (adjusted upward in cases of lower tracking accuracy) during two consecutive zero-crossing intervals, the corresponding time period was flagged as a potential saccade. If a saccade was simultaneously detected by the velocity-based method within the same interval, the event was classified as a saccade. For velocity-based detection, we used a λ of 5 and a minimum saccade duration of 6 ms (i.e., three data samples). To avoid counting fragmented events and reduce false separations, saccadic events were merged if they occurred within 10 ms (i.e., five data samples) of one another. Saccade parameters (e.g., saccade onset, amplitude, peak velocity, etc.) were extracted from the velocity-based detection algorithm applied to the raw data, after co-registering the detected events with those identified using the acceleration-based approach.

#### Exclusion of trials from analysis

##### Experiment 1

We excluded saccades (not entire trials) from all analyses if they occurred during the first 500 ms of the fixation period; participants had been instructed to maintain fixation until the target occluded the fixation dot, and only then begin pursuit. Saccade rates were calculated based on this filtered data. For our analyses of visual and saccade sensitivity, we additionally excluded trials in which more than one catch-up saccade was detected. This was done to ensure a reliable estimation of hit and false alarm rates, as the presence of multiple saccades made it unclear whether—and in response to which event—participants provided their response. We also excluded trials in which a replayed catch-up saccade could have rendered the stimulus visible and in which the participant generated at least one (additional) catch-up saccade.

In Experiment 1, participants completed on average 1566 ± 81 trials. Broken down into valid trials per condition, and after applying all exclusion criteria, participants contributed on average 105 ± 2 trials for the no-stimulus condition (per each target velocity), 209 ± 5 trials for the replay condition, and 208 ± 6 trials for the active condition.

##### Variations in Experiments 2

In Experiment 2, all exclusion criteria were identical to the first experiment. Here, participants completed 2038 ± 98 valid trials, 35 ± 1 trials for the no-stimulus condition (per each combination of target velocity and session), 34 ± 1 trials for the replay condition, and 102 ± 2 trials for the active condition.

##### Variations in Experiments 3

Exclusion criteria in Experiment 3 were identical to the previous two experiments and participants completed on average 1946 ± 92 valid trials; 82 ± 2 trials for the no-stimulus condition (per each combination of eye movement instruction and target velocity), 121 ± 2 trials for the replay condition, and 121 ± 2 trials for the active condition.

### Statistical analysis

#### General approach

All analysis followed a dual frequentist-Bayesian approach: Repeated-measures ANOVAs (rmANOVAs) were used to test for significant effects, using the rstatix package in R (Kassambara, 2023). The package tests for sphericity and applies the Greenhouse-Geisser correction when needed. For clarity of presentation, we report these corrections explicitly only when they change the interpretation of a result. To corroborate findings, we performed Bayesian model comparisons using the BayesFactor package in R (Morey & Rouder, 2012; Rouder, Speckman, Sun, Morey, & Iverson, 2009), applying the default Cauchy prior $\left(r=\sqrt{2}/2\approx 0.707\right)$, and contrasting models with the relevant fixed effects against participant-only null models. This approach allowed us to quantify the relative evidence for competing models, providing complementary information to frequentist significance testing and supporting the interpretation of null or weak effects. Pre-registered predictions for each analysis are listed as Table S0 in the Supplementary Material S0: Pre-registered hypotheses and predicted outcomes.

#### Visual sensitivity to intra-saccadic stimulation

##### Eye movement generation

We first estimated observers’ visual sensitivity to the stimulus in all three experiments by examining their responses to the first question asked after each trial: “Did you perceive a STIMULUS FLASH?” Individual hit rates (HIR) were calculated based on affirmative responses in trials in which a stimulus was present. Similarly, individual false alarm rates (FAR) were calculated based on affirmative responses in trials without a stimulus. To assess the effect of eye movement generation and stimulus visibility on visual sensitivity, HIRs were calculated separately for trials with and without generated and replayed catch-up saccades. Due to the low number of false alarms, FARs were calculated separately for generated and replayed eye movements, but irrespective of whether an eye movement was actually generated. In Experiments 2 and 3, HIRs and FARs were additionally computed by session (Experiment 2) or by eye movement type (intended vs. unintended; Experiment 3). All rates were calculated individually for each observer. Sensitivity was then computed as:

$$d^{'}=z\left(HIR\right)-z\left(FAR\right)$$

We computed average sensitivity indices for trials without (detected) saccade as a descriptive measure of visual sensitivity under pursuit-only conditions and compared these to trials with saccades to examine the effect of saccade generation. To determine whether visual sensitivity differed between generated and replayed saccades, we conducted rmANOVAs with stimulus condition (generated vs. replayed) as a within-subject factor, additionally including session (Experiment 2) or saccade type (intended vs. unintended; Experiment 3) as second within-subject factors when applicable. Bayesian model comparisons included stimulus condition and saccade generation as fixed effects, with participant as a random effect and additionally session (Experiment 2), and saccade type (Experiment 3) where applicable.

##### Effect of target velocity

To assess the effect of target velocity on stimulus visibility, we repeated this analysis separately for each stimulus condition and target velocity (Experiment 1: 6, 9, 12 dva/s; Experiments 2 and 3: 3, 6, 9 dva/s). This was done separately from the main analysis, as data were insufficient for some participants to robustly estimate sensitivity for each velocity level across all conditions. Target velocity effects were evaluated via separate one-way rmANOVAs and complementary Bayesian analyses, contrasting models with target velocity against participant-only null models, for each experiment and catch-up saccade condition.

##### Eye movement kinematics

Because visual sensitivity likely depends on how well the stimulus is stabilized on the retina, we examined sensitivity as a function of retinal velocity during catch-up saccades. Retinal velocity was calculated by subtracting the fixed phase shift speed from each catch-up saccade's peak velocity, with positive values indicating that eye movement and phase shift directions were identical and negative values indicating that they were oriented in opposite directions. We categorized retinal velocities below 30 dva/s as “low” and those above 30 dva/s as “high.” In Experiment 1, HIR and FAR were calculated as in previous analyses. In Experiments 2 and 3, however, the two stimuli were always presented in opposite directions, so we added a second response question during the response phase: participants were asked which stimulus they had seen (i.e., the one above or below the gaze trajectory). A hit was defined as a report of the stimulus location for which the retinal velocity was closer to zero, while a false alarm was defined as a report of the opposite stimulus (i.e., the one for which retinal velocity was farther from zero). Visual sensitivity was then calculated as before, separately for trials with high and low retinal velocities. To test for effects of retinal stabilization, we conducted separate two-way rmANOVAs for each experiment, with retinal velocity (high vs. low) and stimulus condition (generated vs. replayed saccades) as within-subject factors. Because of missing data in at least one condition combination, five participants in Experiment 2 and two participants in Experiment 3 were excluded from this analysis. Complementary Bayesian analyses were conducted for each experiment.

#### Motor control of catch-up saccades

##### Saccade rates

To assess motor control, we analyzed saccade rates, calculated as the number of saccades divided by the number of trials, and normalized by the average trial duration. Saccade rates were analyzed separately for each experiment and calculated separately based on stimulus presence (present vs. absent), target velocity, session (Experiment 2), and saccade type (intended vs. unintended; Experiment 3). We conducted a two-way rmANOVA with the within-subject factors stimulus presence (present vs. absent) and target velocity for Experiment 1, and separate three-way rmANOVAs including session (Experiment 2) or saccade type (intended vs. unintended; Experiment 3). Complementary Bayesian model comparisons included stimulus presence and target velocity as fixed effects and participant as a random effect. The models for Experiments 2 and 3 additionally contained session (Experiment 2) and saccade type (intended vs. unintended; Experiment 3). One participant had missing data in a single condition combination (highest target velocity in one session) in Experiment 2. This participant was excluded from the rmANOVA but retained in the Bayesian model comparison, which accommodates unbalanced data.

##### Saccade latencies

To examine whether participants showed a more subtle form of training effect beyond changes in saccade rate over time, we additionally calculated the latency of the first saccade in each trial and condition, investigating whether participants were able to delay saccade initiation (i.e., withhold a saccade until later in the trial). Because stimulus presence had no effect on saccade rate and we had no preregistered predictions regarding its influence on latency, we excluded this factor from the analysis. Latencies were therefore calculated separately for target velocity, as well as for session (Experiment 2) and saccade type (intended vs. unintended; Experiment 3). We conducted one-way rmANOVAs with target velocity as the sole within-subject factor for Experiment 1, and separate two-way rmANOVAs that additionally included target velocity and either session (Experiment 2) or saccade type (intended vs. unintended; Experiment 3). Complementary Bayesian model comparisons included target velocity as a fixed effect and participant as a random effect, as well as session (Experiment 2) and saccade type (Experiment 3) when applicable. As in our analysis of saccade rate, one participant was excluded from the rmANOVA for Experiment 2 due to missing data but was retained in the Bayesian model comparison, as the model was able to converge.

#### Eye movement sensitivity

Lastly, to assess observers’ awareness of their catch-up saccades, we calculated saccade sensitivity—defined as the ability to judge whether a saccade had been generated in the preceding trial. We analyzed participants’ responses to the question “Do you think you generated a CATCH-UP SACCADE?” A “yes” response in a trial with an actual catch-up saccade was classified as a hit; the same response in a trial without a saccade was classified as a false alarm. Sensitivity was computed as before. This analysis was performed separately for stimulus-present and stimulus-absent trials. Crucially, to control for the influence of (trans-saccadic) visual information—that is, to ensure that saccade detection was not merely driven by visual detection of the stimulus—we adjusted the trial selection for stimulus-present conditions: HIRs were based only on trials in which a generated saccade could have rendered the stimulus visible. FAs, in contrast, were based on replay trials—those without a generated saccade, but in which the replay of a previously generated catch-up saccade could have similarly rendered the stimulus visible. We conducted an additional analysis applying the trial split separately for each target velocity level. We conducted a one-way rmANOVA with stimulus presence (present vs. absent) as a within-subject factor in Experiment 1, a two-way rmANOVA with stimulus presence and session in Experiment 2, and with stimulus presence and saccade type (intended vs. unintended) in Experiment 3. Bayesian model comparisons: In Experiment 1, the model included stimulus presence as a fixed effect and participant as a random effect, and additionally session (Experiment 2), and saccade type (Experiment 3) where applicable.

To test for the effect of target velocity, we repeated the analyses including target velocity as a within-subject factor in Experiment 1, and replacing session or saccade type with it in Experiments 2 and 3. We excluded one participant from the target velocity analysis in Experiment 1, and two participants from the equivalent analysis in Experiment 2. They were excluded from both the rmANOVA and Bayesian model comparisons because the models failed to converge.

### Data and code availability

The preregistration, data, and analysis code of all three experiments have been deposited at the Open Science Framework (OSF) and are publicly available

• Experiment 1: Preregistration: https://osf.io/dtxu5/.
Data and analysis script: https://osf.io/mhfke/.

• Experiment 2: Preregistration: https://osf.io/u7gj4/.
Data and analysis script: https://osf.io/hcf9a/.

• Experiment 3: Preregistration: https://osf.io/w7rtq/.
Data and analysis script: https://osf.io/qbpkz/.

## Results

### Confirmatory analysis: Stimulus efficacy

#### Robust visual sensitivity to intra-saccadic stimulation

**Figure 4.**
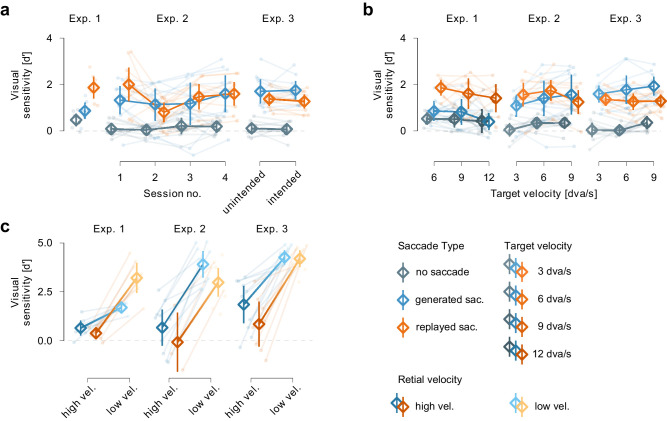
Visual sensitivity depends on saccade generation and their kinematics, but not on target velocity. (**a**) Visual sensitivity to the stimulus in trials without a catch-up saccade, and with either a generated or replayed catch-up saccade. Results are additionally plotted separately across sessions (Experiment 2) to assess whether the stimulus could have the intended training effect over time. Sensitivity is also shown by saccade type (intended vs. unintended; Experiment 3), to determine whether explicitly instructing participants to either generate a saccade or maintain pursuit influenced stimulus perception. (**b**) Visual sensitivity plotted as a function of target velocity (3, 6, 9, and 12 dva/s, depending on the experiment). (**c**) Visual sensitivity as a function of retinal velocity, categorized into high (> 30 dva/s) and low (< 30 dva/s) velocity bins. Error bars represent 95% confidence intervals.

To assess whether the stimulus was visible in the absence of saccades, we calculated visual sensitivity in pursuit-only trials. In Experiment 1, sensitivity was reliably above zero (d′ = 0.47 ± 0.28; Figure 4a). In contrast, sensitivity was close to zero in Experiments 2 and 3 (Experiment 2: d′ = 0.08 ± 0.25; Experiment 3: d′ = 0.09 ± 0.17; Figure 4a).

Across all experiments, visual sensitivity was substantially higher in trials with a generated or replayed catch-up saccade (Experiment 1: d′ = 1.48± 0.33; Experiment 2: d′ = 1.55 ± 0.56; Experiment 3: d′ = 1.43 ± 0.25; Figure 4a). To examine whether stimulus visibility was influenced by stimulus condition (generated vs. replayed saccade), session (Experiment 2), or saccade type (Experiment 3), we conducted additional rmANOVAs for each experiment: In Experiment 1, a one-way rmANOVA revealed a significant main effect of stimulus condition (*F*(1, 7) = 9.74, *p* = 0.017, BF_10_ = 30.77), with higher sensitivity for replayed (d′ = 1.87 ± 0.47) than for generated saccades (d’ = 0.86 ± 0.35; Figure 4a).

In Experiment 2, a two-way rmANOVA found no significant difference in sensitivity between replayed (d′ = 1.47 ± 0.43) and generated (d′ = 1.31 ± 0.70) saccades (*F*(1, 9) = 0.86, *p* > 0.250). However, there was a significant effect of session (*F*(3,27) = 4.61, *p* = 0.010) and a significant interaction between session and stimulus condition (*F*(3, 27) = 6.60, *p* = 0.002; Figure 4a), reflecting a notable drop in sensitivity in the second session, especially for replayed saccades. Bayesian model comparison favored a model including only session (BF_10_ = 18.90), while models incorporating stimulus condition or their interaction were less supported (BF ≤ 0.66 relative to the model with session), consistent with the rmANOVA results.

In Experiment 3, we found a significant effect of stimulus condition (*F*(1, 9) = 6.46, *p* = 0.032), with slightly higher sensitivity for generated (d′ = 1.73 ± 0.41) than for replayed saccades (d′ = 1.32 ± 0.29; Figure 4a). As expected, we found no significant effect of saccade type (intended vs. unintended; *F*(1,9) = 0.05, *p* > 0.250) and no interaction (*F*(1, 9) = 1.05, *p* > 0.250). These results were again supported by the Bayesian analysis, which favored a model including only stimulus condition (BF_10_ = 17.19), whereas models including saccade type or their interaction received substantially less support (BF ≤ 0.29).

#### No reliable effect of target velocity on stimulus visibility

We examined the effect of target velocity on stimulus visibility in separate analyses by calculating separate one-way rmANOVAs for every combination of experiment, saccade, and stimulus condition. Across experiments, target velocity did not significantly affect visual sensitivity in trials without saccadic eye movements (all *p*s ≥ 0.066; Figure 4b). Bayesian analysis likewise indicated no meaningful effect of target velocity on visual sensitivity in trials without catch-up saccades, with models including target velocity receiving little support (BF_10_ ≤ 2.03). Although we did find significant effects for generated saccades in Experiment 1 (*F*(2, 14) = 4.11, *p* = 0.039), and for replayed saccades in Experiment 2 (*F*(2, 16) = 4.30, *p* = 0.032; all remaining *p*s ≥ 0.106; Figure 4b), they were not supported by our Bayesian analysis (BF_10_ ≤ 0.50), suggesting they were not robust.

#### Stimulus perception depends on matching saccade kinematics

To determine whether visual sensitivity depended on the degree of retinal stabilization of the stimulus, we analyzed sensitivity based on retinal stimulus velocity. Retinal velocities were categorized as low (<30 dva/s) or high (>30 dva/s) depending on the combined velocity of eye movement and stimulus on the retina. We conducted individual two-way rmANOVAs for each experiment, with retinal velocity (low vs. high) and stimulus condition (generated vs. replayed) as factors, to assess whether the effect of retinal motion differed between eye movement types. In Experiment 1, we found a strong effect of retinal velocity (*F*(1, 7) = 76.68, *p* < 0.001), with sensitivity being higher for low (d′ = 2.45 ± 0.45) than for high retinal velocities (d′ = 0.52 ± 0.25; Figure 4c). We also observed a significant main effect of stimulus condition (*F*(1, 7) = 8.04, *p* = 0.025), showing greater sensitivity for replayed (d′ = 1.80 ± 0.46) compared to generated saccades (d′ = 1.17 ± 0.23). Additionally, a significant interaction (*F*(1, 7) = 107.1, *p* < 0.001) indicated that replayed saccades benefitted more from low retinal velocities than generated catch-up saccades (Figure 4c). These results were further supported by Bayesian model comparison, which strongly favored a model including stimulus condition, retinal velocity, and their interaction (BF_10_ = 4.01 × 10^8^), whereas models excluding the interaction or any main effect were much less supported (BF_10_ ≤ 1.01 × 10^6^).

In Experiment 2, we again observed a strong effect of retinal velocity (*F*(1, 4) = 57.83, *p* = 0.002), with no significant effect of stimulus condition (*F*(1, 4) = 2.87, *p* = 0.166) and no interaction (*F*(1, 4) = 0.02, *p* > 0.250), suggesting that visual sensitivity was primarily driven by the degree of retinal stabilization. Our Bayesian model comparison strongly favored the model including retinal velocity and stimulus condition (BF_10_ = 1.42 × 10^7^). Models including the interaction or excluding stimulus condition or retinal velocity were substantially less supported (BF ≤ 0.47 relative to the best model), supporting that retinal velocity was driving visual sensitivity.

Experiment 3 showed a nearly identical pattern: a strong effect of retinal velocity (*F*(1, 7) = 31.31, *p* < 0.001), in the absence of an effect of stimulus condition (*F*(1, 7) = 2.80, *p* = 0.138), and no interaction (*F*(1, 7) = 4.3, *p* = 0.077), confirming that low retinal velocity enhanced stimulus visibility. The results of our Bayesian model comparison once again confirmed these findings, which strongly favored a model that included retinal velocity (BF_10_ = 3.09 × 10^7^), whereas models incorporating stimulus condition or the interaction received less support (BF ≤ 0.75 relative to the model).

### Main result I: Voluntary motor control

#### Saccade rates reveal limited voluntary motor control for catch-up saccades

**Figure 5.**
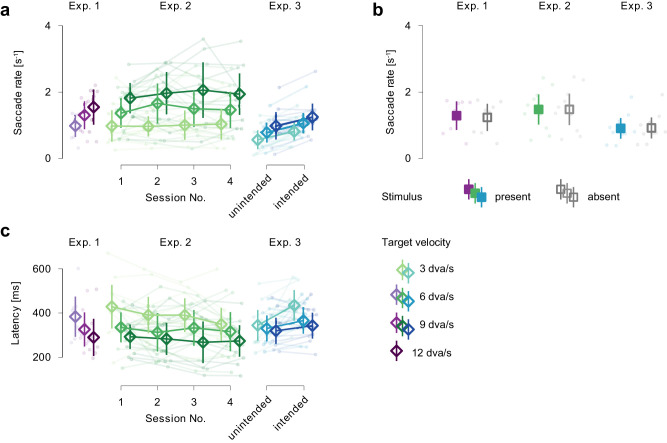
Saccade rate increases with target velocity in all experiments and decreases with explicit pursuit instruction (Experiment 3). (**a**) Saccade rate as a function of target velocity (all experiments), how it develops with training (e.g., session number; Experiment 2), and following the explicit instruction to pursue or saccade (e.g., unintended vs. intended, Experiment 3). (**b**) Saccade rate as a function of stimulus presentation for all experiments. (**c**) Saccade latency displayed using the same structure as in a. In all panels: Error bars represent 95% confidence intervals.

To assess the extent of volitional control our observers exerted over catch-up saccade generation, we examined saccade rates (Experiment 1), their evolution over time while participants were instructed to suppress saccades (Experiment 2), the saccade type (intended vs. unintended saccades; Experiment 3), as well as the effect of stimulus presence (present vs. absent). A two-way rmANOVA in Experiment 1 revealed a significant main effect of target velocity (*F*(2, 14) = 28.1, *p* < 0.001), with saccade rates increasing as target velocity increased (6 dva/s = 0.98 ± 0.33 s^−^^1^; 9 dva/s = 1.30 ± 0.42 s^−^^1^; 12 dva/s = 1.55 ± 0.53 s^−^^1^; Figure 5a). Neither the main effect of stimulus presence (*F*(1, 7) = 1.78, *p* = 0.224; Figure 5b) nor the interaction between stimulus presence and velocity reached significance (*F*(2, 14) = 3.17, *p* = 0.073), suggesting that the visibility of the stimulus did not facilitate suppression of catch-up saccades. A Bayesian model comparison, conducted to corroborate these null-results, provided very strong evidence for an effect of target velocity over the null model (BF_10_ = 1.0 × 10^9^), while the model with only stimulus presence was not supported (BF_10_ = 0.30). Including both main effects slightly reduced model evidence (BF_10_ = 5.0 × 10^8^) and adding their interaction further decreased support (BF = 0.31 relative to the model comprising only the two main effects), providing no evidence for an interaction between stimulus presence and target velocity.

This finding was confirmed by the data from Experiment 2: We observed a comparable increase in saccade rate with target velocity (3 dva/s = 1.00 ± 0.18 s^−^^1^; 6 dva/s = 1.50 ± 0.23 s^−^^1^; 9 dva/s = 1.94 ± 0.28 s^−^^1^; Figure 5a), which was confirmed as statistically significant by a three-way rmANOVA (*F*(2, 16) = 22.42, *p* < 0.001). Neither the effect of stimulus presence (*F*(1,8) = 0.002, *p* > 0.250 Figure 5b) nor that of session (*F*(3, 24) = 0.64, *p* > 0.250) was significant, suggesting that stimulus presentation did not help participants suppress their catch-up saccades, nor did repeated exposure across sessions lead to a reduction in catch-up saccade generation over time. Additionally, none of the interactions were significant (all *p*s ≥ 0.224). Bayesian model comparison yielded strong evidence for models including target velocity (BF_10_ = 1.2 × 10^29^), whereas models that excluded target velocity while including stimulus presence (BF_10_ ≤ 0.14) or session (BF_10_ ≤ 0.05) were not supported. Even the most complex interaction models were decisively less supported than the model solely including target velocity (BF_10_ = 0.14), providing no indication of added explanatory value by factors beyond target velocity alone. An analysis of potential long-term training effects comparing expert and naïve participants) is appendant to this manuscript (see Supplementary Materials S2: Observer groups in Experiment 2).

In Experiment 3, we again observed a significant effect of target velocity on saccade rate. Saccade rates increased significantly with increasing target speed (*F*(2, 18) = 16.78, *p* < 0.001; 3 dva/s = 0.69 ± 0.19 s^−^^1^; 6 dva/s = 0.92 ± 0.21 s^−^^1^; 9 dva/s = 1.12 ± 0.27 s^−^^1^; Figure 5a). Importantly, our three-way rmANOVA revealed a significant difference between intended and unintended saccades (*F*(1, 9) = 16.41, *p* = 0.003), indicating that participants generated significantly fewer saccades when explicitly instructed to pursue (mean = 0.77 ± 0.13 s^−^^1^) compared to when instructed to make a saccade (mean = 1.05 ± 0.12 s^−^^1^). Stimulus presence, on the other hand, did not significantly affect saccade rate (*F*(1, 9) = 0.94, *p* > 0.250; Figure 5b). This demonstrates again that saccade-contingent visual feedback did not facilitate volitional control over catch-up saccades. We also did not observe any significant interactions between other factors (all *p*s ≥ 0.182). Like in the previous two experiments, Bayesian model comparison showed overwhelming evidence favoring models including target velocity (BF_10_ ≥ 1.2 × 10^9^ for all such models). Unlike in the previous experiments, in Experiment 3 we observed the highest Bayes factor for the model including saccade type as well as target velocity (BF_10_ = 7.57 × 10^18^). Models excluding target velocity or including only stimulus presence or saccade type had substantially lower support (BF_10_ ≤ 1.2 × 10^5^). Adding interactions involving saccade type, stimulus presence, and target velocity consistently decreased model evidence, indicating no meaningful contribution of these interaction terms beyond the main effects of target velocity or saccade type. Details on how saccade distance, direction, and cue timing affected saccade rate and amplitude in the instructed saccade trials are provided in the Supplementary Material (see Supplementary Materials, S3: Instructed saccade conditions in Experiment 3).

#### Saccade latencies support limited voluntary motor control

We conducted a secondary analysis on the latency of the first saccade in each trial, as saccade frequency might have been too coarse to detect subtle effects of stimulus presence, training, or intention. Across three separate rmANOVAs, we consistently found significantly decreasing saccade latencies with increasing target speeds in all experiments (all *p*s ≤ 0.003; Figure 5c), indicating a faster need for catch-up saccades as target velocity rises. Stimulus presence was consistently non-significant (all *p*s ≥ 0.227), as was training in Experiment 2 (*p* > 0.250). However, saccade type in Experiment 3 had a significant effect on saccade latency (*p* = 0.048), with a significant interaction between target velocity and instruction emerging only in this experiment (*p* = 0.004). Bayesian model comparisons for each experiment helped further evaluate the contributions of target velocity (across all experiments) and session (Experiment 2) or saccade type (Experiment 3) on saccade latency. In Experiment 1, the model including only target velocity and participant received the strongest support (BF_10_ = 9.6 × 10^6^) with all other models receiving substantially less support (BF ≤ 0.28). In Experiment 2, the same model was again best supported (BF_10_ = 1.77 × 10^14^), with less evidence for the model that additionally included session (BF = 0.30). In Experiment 3, however, the comparison determined that the model including target velocity, saccade type, and their interaction was best supported (BF_10_ ≥ 3.1 × 10^7^), while the simpler models including only saccade type (BF_10_ = 1.9 × 10^3^) or target velocity (BF_10_ = 1.02 × 10^3^) received substantially less support. These results suggest that while target velocity was a consistent predictor across experiments, additional variance was explained by session in Experiment 2 and, even more substantially, by saccade type in Experiment 3.

### Main result II: Saccade awareness

#### Minimal awareness of catch-up saccades

**Figure 6.**
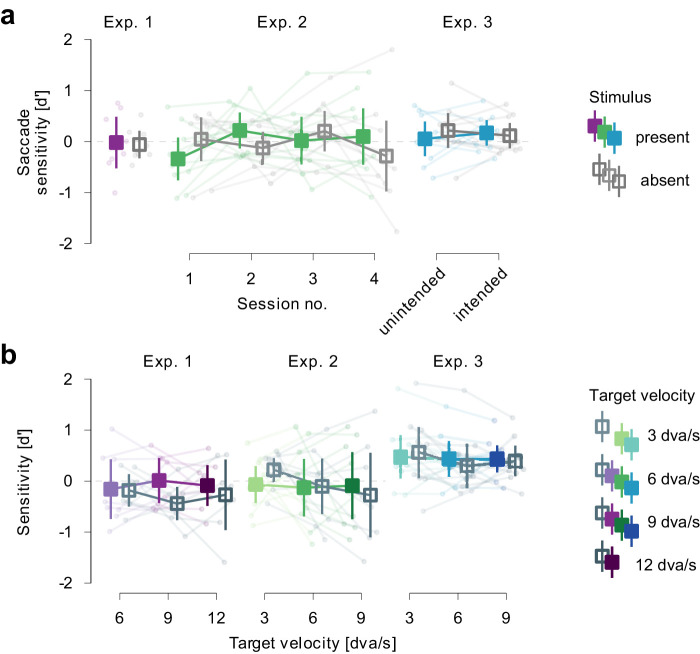
Low saccade sensitivity does not benefit from saccade-contingent feedback, cannot be trained (Experiment 2) and does not improve when movements are explicitly instructed (Experiment 3). (**a**) Saccade sensitivity as a function of stimulus presence (all experiments), its development over time (e.g., session number; Experiment 2), and following the instruction to pursue or make a catch-up saccade (e.g., unintended vs. intended, Experiment 3). (**b**) Saccade sensitivity as a function of stimulus presentation for all experiments and target velocities. In all panels: Error bars represent 95% confidence intervals.

We examined awareness of catch-up saccades during pursuit by analyzing observer's saccade sensitivity: their ability to distinguish between trials with and without a catch-up saccade. To evaluate whether saccade-contingent visual feedback provided by our stimulus enhanced detection, we analyzed this separately for trials with and without the stimulus. In Experiment 1, saccade sensitivity was close to zero, whether the stimulus was present (d′ = −0.02 ± 0.50) or absent (d′ = −0.06 ± 0.27). A one-way rmANOVA revealed no significant effect of stimulus presence (*F*(1,7) = 0.08, *p* > 0.250; Figure 6a). A Bayesian model comparison corroborated this result, with the model including stimulus presence receiving less support than the null model (BF_10_ = 0.43), indicating no evidence for an effect of stimulus presence. For a more detailed breakdown of hit and false alarm rates across experiments, see Supplementary Material S4: A closer look at saccade sensitivity: Hit and false alarm rates across experiments.

Similarly, in Experiment 2, we found sensitivity equally close to zero irrespective of stimulus presence (present: d′ = 0.00 ± 0.34; absent: d′ = −0.04 ± 0.37; Figure 6a). A two-way rmANOVA, which included session number to assess potential training effects, revealed no significant main effects of stimulus presence (*F*(1, 9) = 0.06, *p* > 0.250) or session (*F*(3, 27) = 0.93, *p* > 0.250). Despite a significant interaction (*F*(3, 27) = 4.40, *p* = 0.012), indicating that the effect of stimulus presence varied across sessions, we conclude from this analysis that neither saccade-contingent feedback nor training reliably improved detection performance. These findings were again corroborated by a Bayesian model comparison, which showed that all models—including those with stimulus presence or session—received less support than the null model (BF_10_ ≤ 0.25).

Sensitivity in Experiment 3 was similarly low, with values near zero for both stimulus-present (d′ = 0.11 ± 0.24) and stimulus-absent trials (d′ = 0.16 ± 0.25; Figure 6a). Finally, to determine whether movement intention (potentially combined with feedback) affected awareness of saccades, we conducted a two-way rmANOVA with stimulus presence and saccade type (intended vs. unintended) as factors. Neither the main effects (stimulus presence: (*F*(1, 9) = 0.18, *p* > 0.250; saccade type *F*(1, 9) = 0.00, *p* > 0.250) nor their interaction (*F*(1, 9) = 1.86, *p* = 0.206; Figure 6a) were significant, suggesting, once again, low saccade sensitivity that remained largely unaffected by feedback and movement intention. In Bayesian model comparison, all models received less support than the null model (BF_10_ ≤ 0.34), regardless of whether they included stimulus presence, saccade type, or their interaction.

#### Target speed does not modulate saccade awareness; active intent may

To examine whether target velocity influenced saccade sensitivity, we conducted separate two-way rmANOVAs for each experiment, with stimulus presence (absent vs. present) and target velocity as factors (6, 9, 12 dva/s in Experiment 1; 3, 6, 9 dva/s in Experiments 2 and 3). Across all experiments, we found no significant main effects or interactions (all *p*s ≥ 0.160, BF_10_ ≤ 0.85), indicating that variations in target velocity did not affect saccade detection performance, regardless of stimulus presence (Figure 6b). To compare saccade sensitivity across experiments, we conducted a one-way ANOVA with experiment as the sole between-subjects factor. Although the analysis revealed no significant differences between experiments (*F*(2, 25) = 1.91, *p* = 0.169, BF_10_ = 0.70), descriptive statistics showed that saccade sensitivity—while generally low—was significantly above zero in Experiment 3 (Experiment 3: d′ = 0.45 ± 0.29), compared to non-significant values in the other two experiments (Experiment 1: d′ = 0.02 ± 0.39; Experiment 2: d′ = 0.45 ± 0.47; Figure 6b).

## Discussion

### General discussion

In a series of three experiments, we examined whether observers could control catch-up saccades during pursuit and whether they were aware of these brief ballistic eye movements. Our results reveal a clear dissociation: although target velocity and explicit instructions modulated saccade rates—suggesting a limited degree of low-level control—awareness of catch-up saccades remained minimal and resistant to both perceptual and intentional modulation.

Our confirmatory analysis of visual sensitivity reveals that sensitivity was low in the absence of a saccade and increased when a saccade was generated, confirming the effectiveness of saccade-contingent visual feedback (Figure 4a). Moreover, visibility depended on the degree of retinal stabilization provided by the saccade, confirming that the stimulus provides specific rather than generic feedback about saccade execution (Figure 4c). Together, these findings demonstrate that the stimulus becomes visible saccade-contingently—whether generated or replayed—providing immediate visual feedback about saccade execution.

Across all analyses, saccade rates consistently increased with target velocity but decreased when saccades were unintended, suggesting that our trial-by-trial instructions helped participants suppress saccades and that a certain modest level of control was possible (Figure 5a). However, the absence of effects from stimulus feedback and training demonstrates that low-level factors—such as target speed and explicit instructions—drive this modulation rather than high-level volitional control, a conclusion further supported by our secondary analysis of saccade latencies (Figure 5c).

Saccade sensitivity remained consistently low, unaffected by stimulus presence, target velocity, training, or movement intention (Figure 6a). Unlike microsaccade awareness (Klanke, Ohl, & Rolfs, 2025b), catch-up saccade awareness was unaffected by intention—despite intention successfully modulating voluntary control over catch-up saccades in the current study. These findings suggest that awareness of catch-up saccades during pursuit is minimal and resistant to both perceptual and intentional modulation.

### Voluntary control of catch-up saccades

Although participants were able to exert some voluntary control over their catch-up saccades, as indicated by reduced rates when explicitly (i.e., visually) prompted to suppress them, this ability was markedly limited: control did not improve with training (Figure 5a), and participants were unable to use immediate saccade-contingent visual feedback to further reduce saccade rates (Figure 5b). Instead, saccade rate was most consistently modulated by target velocity, with more saccades occurring at higher speeds (Figure 5a). This pattern indicates that catch-up saccade generation is primarily driven by task demands—potentially to maintain foveation on a fast-moving target (cf., Heinen, Potapchuk, & Watamaniuk, 2016) or to correct for low-level position errors (i.e., “retinal slip”; Daye, Blohm, & Lefevre, 2014; Mcilreavy, Freeman, & Erichsen, 2019; Schröder, Keidel, Trautner, Radbruch, & Ettinger, 2023)—rather than volitional control. Interestingly, the reduction in saccade rates following the suppression cue may similarly reflect the influence of task dynamics rather than the prompt itself. By presenting this cue visually on every trial, we likely allowed participants to adjust their behavior indirectly, responding to the visual information inherent in the task rather than through a direct exertion of will. Further support for this interpretation comes from our analysis of intended catch-up saccades in Experiment 3. We found that participants adjusted their saccade rates primarily based on temporal aspects of the task, making more saccades when the go-cue appeared earlier, and only to a lesser extent in response to spatial factors. In contrast, they modulated saccade amplitude mainly according to spatial features—such as the distance and direction of the instructed saccade—while cue timing had little to no effect (see Supplementary Materials S3: Saccade parameters in response to target manipulations in Experiment 3). These results suggest that participants adapted their eye movements in response to the visually presented goal (i.e., the target position), corroborating the idea that saccades can be modulated voluntarily during pursuit. Together, these findings demonstrate that low-level factors primarily drive catch-up saccade generation during pursuit and suggest that although conscious, top-down control over these movements is possible, it remains limited.

### Awareness of catch-up saccades

Although humans can retain some information about their past saccades, conscious awareness of their own eye movements remains poor. Recognition of past fixations is often inaccurate, with observers sometimes confusing their own fixations with those of others or even recalling fixations that did not occur (Marti, Bayet, & Dehaene, 2015; Võ, Aizenman, & Wolfe, 2016). Awareness remains low even with online gaze feedback (Kok, Aizenman, Võ, & Wolfe, 2017) or offline gaze animations after inspection (Van Wermeskerken, Litchfield, & Van Gog, 2018). Some studies suggest that observers can estimate their saccade latencies (Vencato & Madelain, 2020), but this ability is generally limited and influenced by biases such as chronostasis (Brown & Rothwell, 1997; Yarrow, Haggard, Heal, Brown, & Rothwell, 2001) and pre-dating mechanisms supporting visual stability (Hunt & Cavanagh, 2009; Nörenberg, Schweitzer, & Rolfs, 2025; Yarrow, Whiteley, Haggard, & Rothwell, 2006). Although informative, these studies mostly focus on “regular” saccades and use metrics for movement awareness that differ from our approach. Interestingly, our previous work on microsaccades—much smaller eye movements than typical saccades—showed that while participants have some level of conscious awareness of these movements (Klanke et al., 2025b), we found no evidence that they are accompanied by a measurable feeling of agency (Klanke et al., 2025a), suggesting that although microsaccades can be detected introspectively, they are not experienced in rich detail.

Consistent with this limited awareness, participants in our study showed little conscious access to their catch-up saccades, despite some volitional control: Across all experiments, awareness of these saccades remained low, regardless of stimulus presence, training, movement intention (Figure 6a), or eye-movement expertise (see Supplementary Materials S2: Observer groups in Experiment 2). This dissociation suggests that although catch-up saccades can be modulated intentionally to some extent, they remain largely inaccessible to conscious monitoring, pointing to a functional separation between oculomotor control and introspective access. This is particularly surprising given the comparatively large amplitudes of the catch-up saccades in our data—which, depending on the experiment and condition, averaged around 1.5 dva. In contrast, a study by Klanke et al. (2025a), which investigated awareness of microsaccades, found that these much smaller eye movements—only 1 dva or less—were nonetheless sometimes accessible to introspection. A possible explanation for the low awareness of catch-up saccades in the present study is that participants’ attention was focused on the ongoing smooth pursuit—potentially masking awareness of saccades embedded within it. The initiation (Hashimoto, Suehiro, Kodaka, Miura, & Kawano, 2003) and control (Lovejoy, Fowler, & Krauzlis, 2009) of pursuit are closely linked to the deployment of attentional resources, as maintaining accurate pursuit requires continuously directing visuospatial attention to the target's predicted position. This suggests that pursuit and the processing of visual information rely on a shared attentional mechanism (Chen, Holzman, & Nakayama, 2002; Khurana & Kowler, 1987). In this view, the binding of visuospatial attention to the pursuit target creates the illusion of uninterrupted, smooth tracking. Discrete corrective movements like catch-up saccades, which might otherwise draw introspective notice, would thereby be rendered introspectively “invisible.” This continuous attentional demand may thus leave insufficient resources for monitoring the internally generated motor signals associated with catch-up saccades. Support for this idea comes from evidence that pursuit performance deteriorates when attention is allocated elsewhere—for example, when participants are engaged in a secondary task (Hutton & Tegally, 2005).

The effortlessness with which catch-up saccades are usually blended into our sense of a fluid, continuous pursuit becomes most apparent when the (predictive) pursuit machinery breaks down. Koerfer, Watson & Lappe (2024) provide a vivid demonstration with their non-rigid moving vortex: during fixation the pattern looks perfectly coherent, yet when observers try to track it, smooth pursuit gain collapses to almost zero and the target can be followed only by a string of catch-up saccades (Koerfer et al., 2024). The attempt to pursue the stimulus thus turns the normally imperceptible catch-up saccades into conspicuous events, with the disruption of perceptual flow allowing awareness of each corrective eye movement.

Interestingly, our data contrasts somewhat with recent findings by Goettker, Locke, Gegenfurtner, & Mamassian (2024), who reported that observers were able to evaluate the accuracy of their combined pursuit and saccadic eye movements when tracking unpredictable targets (for an analysis of the effect of pursuit gain in our manipulations, see Supplementary Material S6: Pursuit gain). However, several differences between the paradigms may help reconcile these results. For one, the task in Goettker et al. emphasized tracking accuracy rather than awareness of movement occurrence, potentially engaging different cognitive processes. Additionally, their paradigm used visual information and performance history (e.g., gaze–target deviation from a visible sinusoidal trajectory and self-comparison to past performance), potentially allowing participants to rely on external visual cues and performance heuristics, rather than direct introspective access to eye movements themselves. Finally, even in Goettker et al.’s study, metacognitive sensitivity for eye movements remained considerably lower than for hand movements, reinforcing the idea that access to oculomotor events is fundamentally constrained—even when conditions favor introspective awareness. Overall, our findings complement those of Goettker et al. by highlighting that, even when some degree of access to catch-up saccades is possible—as their results suggest—conscious awareness of these movements remains limited, particularly when external cues and comparative feedback are minimized.

### Eye movement and stimulus parameters, visual sensitivity, and perception

Our analysis of visual sensitivity revealed that stimuli with rapid temporal phase shifts can selectively target specific eye movement types—such as catch-up saccades—while remaining largely invisible during others, like smooth pursuit (Figures 4a, 4b). Crucial to this selective visibility is a precise alignment between the stimulus properties and the dynamic parameters of the eye movements. Even slight mismatches in frequency, velocity, or timing—whether in the stimulus design or assumptions about the eye movements—can substantially reduce stimulus visibility and thus diminish its effectiveness. In Experiment 1, we assumed a relatively large and fast catch-up saccade profile when designing the stimulus, which led to a mismatch for many participants and allowed the stimulus to become faintly visible even during pursuit-only trials. In contrast, fine-tuning the stimulus to better match individual saccade dynamics in Experiments 2 and 3 effectively eliminated visibility in the absence of saccades and optimized contingent visibility during catch-up saccades. Across all experiments, visual sensitivity was closely tied to retinal stabilization, with low retinal velocities consistently producing greater sensitivity. Our data, hence, demonstrate the importance of calibrating saccade-contingent stimuli to individual eye movement characteristics and emphasize the role of fine-grained sensorimotor tuning in shaping visual perception during movement.

Seeing the stimulus saccade-contingently does not necessarily imply that participants understood the systematic relationship between their eye movements and the perceptual feedback. In Experiment 1, we additionally asked participants how certain they were that a catch-up saccade had caused the stimulus to become visible—if they had previously reported both seeing the stimulus and making a saccade—or, alternatively, how certain they were that the stimulus had not been caused by a saccade—if they reported seeing the stimulus but denied making an eye movement. Our analysis of these responses suggests generally low certainty, with answers clustering around the midpoint of the scale (i.e., the point of highest uncertainty; see Supplementary Figure S1). This indicates that participants were, on average, unable to reliably distinguish between trials in which the stimulus was caused by a saccade and those in which it was not. Although this could in part be driven by the suboptimal stimulus configuration in Experiment 1, we believe it primarily reflects a general ambiguity regarding the connection between saccades and their visual consequences—particularly when alternative perceptual interpretations (i.e., an identical visual event occurring without a saccade, as in the replay condition) are presented alongside the saccade-contingent change in visual perception (see section S1: Causal assignment from Experiment 1 in the Supplementary Materials).

### The role of intention for awareness

In Experiment 3, our goal was to manipulate movement intention by instructing participants either to pursue the target naturally (unintended saccade condition) or to generate a catch-up saccade deliberately (intended saccade condition). It remains an open question whether this truly reflects a change in intention as opposed to a strategic response to task demands or an effect of attention. However, the robust increase in saccade rates in the intended saccade condition compared to the unintended one suggests that the manipulation successfully altered participants’ volitional engagement with their eye movements. Surprisingly, despite this intentional engagement, saccade sensitivity—that is, participants’ awareness of their own catch-up saccades—remained very low, especially in light of recent findings by Klanke et al. (2025a) who reported higher awareness for microsaccades under similar conditions. A supplementary analysis revealed that this disconnect was due to a significant increase in both hit and false alarm rates when saccades were instructed, suggesting that while participants were more responsive overall, they were not more accurate in distinguishing when a saccade had actually occurred (see Supplementary Materials S4: A closer look at saccade sensitivity: Hit and false alarm rates across experiments). Crucially, our data therefore support that our manipulation indeed affected intention rather than simply task strategy or attention: Participants not only followed the instruction to make a saccade as well as they could, but also genuinely believed they had done so—even when they had not. To better understand how intention influenced awareness, we compared saccade sensitivity across all three experiments. Our analysis revealed that while sensitivity was slightly above zero in all three experiments, it was only significantly different from zero in Experiment 3. This aligns with Klanke et al.’s (2025a) finding that intention can enhance awareness for microsaccades, irrespective of whether the microsaccades were intended or unintended. Notably, Experiment 3 included both intended and unintended saccade conditions, whereas the other experiments—particularly Experiment 1, which showed the lowest saccade sensitivity—treated catch-up saccades as spontaneous. The slight increase in awareness observed in Experiment 3 suggests that explicit intention can moderately enhance sensitivity, even though overall awareness remains low. While our manipulation of intention was, hence, likely successful, its effect on saccade awareness was minimal, indicating that conscious access to saccades during ongoing pursuit remains limited even when these movements are voluntarily produced.

### In pursuit of saccade awareness

Smooth pursuit has long been understood as a voluntary eye movement (c.f., Kowler, 2011) that involves both sensory inputs and cognitive influences. Especially Eileen Kowler's influential research highlighted how pursuit can be modulated by cognitive processes like attention (Khurana & Kowler, 1987; Murphy, Kowler, & Steinman, 1975), expectation (Kowler, 1989; Kowler, Martins, & Pavel, 1984; Kowler & Steinman, 1979; Kowler & Steinman, 1981), and learning (Kowler, 1989; Kowler, 2011; Kowler et al., 1984)—in addition to task affordances (Kowler & McKee, 1987). Our findings complement the work by Eileen Kowler and extend it to catch-up saccades. Although our data show that these corrective saccades are predominantly shaped by low-level task demands such as target velocity, we also observed a small but reliable effect of intention on saccade generation—suggesting that motor control of saccades during pursuit is open to top-down modulation and responsive to cognitive influences. While Eileen Kowler did not explicitly address conscious awareness of pursuit eye movements in her research, her seminal findings on anticipatory pursuit support the idea that these movements are not purely reflexive. They can be generated from memorized cues (Santos & Kowler, 2017), are modulated by the expected target motion (Kowler & Steinman, 1979), and have even been linked to motor intention directly (Kowler et al., 2015; Kowler, Rubinstein, Santos, & Wang, 2019). This degree of volitional control implies that participants have access to internal information about their eye movements. In other words, if a movement can be voluntarily guided, it is likely that the person is at least partially aware of the movement as it is being generated, potentially linking anticipatory pursuit to movement awareness. In stark contrast, our research indicates that catch-up saccades almost always escape awareness. While intention may modestly enhance awareness (see previous section: The role of intention for awareness), we consistently found saccade sensitivity near zero across all experiments and conditions. This suggests that, perhaps unlike smooth pursuit, catch-up saccades remain largely inaccessible to conscious monitoring. Together, our findings suggest that while pursuit eye movements and catch-up saccades are tightly linked components of oculomotor behavior, they differ fundamentally in how they interface with voluntary control and awareness. Our work extends Eileen Kowler's research by revealing that voluntary control does not necessarily extend to awareness, even for closely linked eye movement behaviors.

## Conclusions

When we watch koi in a pond, we experience the illusion that they remain at the center of our gaze despite their constant slow coasting. This illusion persists even though our smooth pursuit is far from perfect and is frequently interrupted by catch-up saccades—even when the gaze target moves at low speeds. Our data suggest that catch-up saccade frequency is strongly modulated by target velocity, with more saccades occurring at higher speeds. These saccades are open to conscious motor control—if the observer can exploit dynamic visual information to modulate their eye movements—but remain inaccessible to introspective awareness, even when accompanied by (trans-saccadic) visual transients. This dissociation between control and awareness highlights the reflexive, opaque nature of corrective eye movements and suggests that the visual system favors visual stability over introspective access to the eye movements that enable it: We can follow the koi effortlessly—without ever noticing the corrections our eyes perform along the way.

## Supplementary Material

Supplement 1

## Footnotes

1 Assuming an average coasting velocity of 60–85 mm s^−1^ (c.f., Wu, Yang, & Zeng, 2007) and an observer distance between 2.5 and 10 meters, koi fish travel at approximately 0.3–2 dva/s.

## References

[bib1] Belopolsky, A. V., Kramer, A. F., & Theeuwes, J. (2008). The Role of Awareness in Processing of Oculomotor Capture: Evidence from Event-related Potentials. *Journal of Cognitive Neuroscience,* 20(12), 2285–2297, 10.1162/jocn.2008.20161. 18457508

[bib2] Brainard, D. H. (1997). The Psychophysics Toolbox. *Spatial Vision,* 10(4), 433–436, 10.1163/156856897X00357. 9176952

[bib3] Brown, P., & Rothwell, J. C. E. (1997). Illusions of time. *Abstracts, 27th Annual Meeting,* 23, 1119.

[bib4] Castet, E., & Masson, G. S. (2000). Motion perception during saccadic eye movements. *Nature Neuroscience,* 3(2), 177–183, 10.1038/72124. 10649574

[bib5] Chen, Y., Holzman, P. S., & Nakayama, K. (2002). Visual and cognitive control of attention in smooth pursuit. *Progress in Brain Research,* 140, 255–265, 10.1016/S0079-6123(02)40055-6. 12508595

[bib6] Collewijn, H., Erkelens, C. J., & Steinman, R. M. (1988). Binocular co-ordination of human horizontal saccadic eye movements. *The Journal of Physiology,* 404(1), 157–182, 10.1113/jphysiol.1988.sp017284. 3253429 PMC1190820

[bib7] Cornelissen, F. W., Peters, E. M., & Palmer, J. (2002). The Eyelink Toolbox: Eye tracking with MATLAB and the Psychophysics Toolbox. *Behavior Research Methods, Instruments, & Computers,* 34(4), 613–617, 10.3758/BF03195489. 12564564

[bib8] Daye, P. M., Blohm, G., & Lefevre, P. (2014). Catch-up saccades in head-unrestrained conditions reveal that saccade amplitude is corrected using an internal model of target movement. *Journal of Vision,* 14(1), 12, 10.1167/14.1.12. PMC4523018 24424378

[bib9] De Brouwer, S., Yuksel, D., Blohm, G., Missal, M., & Lefèvre, P. (2002). What Triggers Catch-Up Saccades During Visual Tracking? *Journal of Neurophysiology,* 87(3), 1646–1650, 10.1152/jn.00432.2001. 11877535

[bib10] Deubel, H., & Elsner, T. (1986). Threshold perception and saccadic eye movements. *Biological Cybernetics,* 54(6), 351–358, 10.1007/BF00355540. 3756240

[bib11] Deubel, H., Elsner, T., & Hauske, G. (1987). Saccadic eye movements and the detection of fast-moving gratings. *Biological Cybernetics,* 57(1–2), 37–45, 10.1007/BF00318714. 3620544

[bib12] Engbert, R., & Mergenthaler, K. (2006). Microsaccades are triggered by low retinal image slip. *Proceedings of the National Academy of Sciences,* 103(18), 7192–7197, 10.1073/pnas.0509557103. PMC1459039 16632611

[bib13] Fooken, J., & Spering, M. (2020). Eye movements as a readout of sensorimotor decision processes. *Journal of Neurophysiology,* 123(4), 1439–1447, 10.1152/jn.00622.2019. 32159423 PMC7191514

[bib14] Goettker, A., & Gegenfurtner, K. R. (2021). A change in perspective: The interaction of saccadic and pursuit eye movements in oculomotor control and perception. *Vision Research,* 188, 283–296, 10.1016/j.visres.2021.08.004. 34489101

[bib15] Goettker, A., Locke, S. M., Gegenfurtner, K. R., & Mamassian, P. (2024). Sensorimotor confidence for tracking eye movements. *Journal of Vision,* 24(8), 12, 10.1167/jov.24.8.12. PMC11363210 39177998

[bib16] Harris, D. J., Wilson, M. R., Jones, M. I., De Burgh, T., Mundy, D., Arthur, T., … Vine, S. J. (2023). An investigation of feed-forward and feed-back eye movement training in immersive virtual reality. *Journal of Eye Movement Research,* 15(3), 1–14, 10.16910/jemr.15.3.7. PMC11229047 38978970

[bib17] Hashimoto, K., Suehiro, K., Kodaka, Y., Miura, K., & Kawano, K. (2003). Effect of target saliency on human smooth pursuit initiation: Interocular transfer. *Neuroscience Research,* 45(2), 211–217, 10.1016/s0168-0102(02)00227-4. 12573467

[bib18] Heinen, S. J., Potapchuk, E., & Watamaniuk, S. N. J. (2016). A foveal target increases catch-up saccade frequency during smooth pursuit. *Journal of Neurophysiology,* 115(3), 1220–1227, 10.1152/jn.00774.2015. 26631148 PMC4808105

[bib19] Hunt, A. R., & Cavanagh, P. (2009). Looking ahead: The perceived direction of gaze shifts before the eyes move. *Journal of Vision,* 9(9), 1, 10.1167/9.9.1. 19761334 PMC2766570

[bib20] Hutton, S. B., & Tegally, D. (2005). The effects of dividing attention on smooth pursuit eye tracking. *Experimental Brain Research,* 163(3), 306–313, 10.1007/s00221-004-2171-z. 15654587

[bib21] Kassambara, A. (2023). *rstatix: Pipe-Friendly Framework for Basic Statistical Tests*. https://rpkgs.datanovia.com/rstatix/.

[bib22] Kelly, D. H. (1990). Moving gratings and microsaccades. *Journal of the Optical Society of America A,* 7(12), 2237–2244, 10.1364/JOSAA.7.002237. 2090802

[bib23] Khurana, B., & Kowler, E. (1987). Shared attentional control of smooth eye movement and perception. *Vision Research,* 27(9), 1603–1618, 10.1016/0042-6989(87)90168-4. 3445492

[bib24] Kinder, A., Rolfs, M., & Kliegl, R. (2008). Sequence Learning at Optimal Stimulus–Response Mapping: Evidence from a Serial Reaction Time Task. *Quarterly Journal of Experimental Psychology,* 61(2), 203–209, 10.1080/17470210701557555. 17886161

[bib25] Klanke, J.-N., Ohl, S., & Rolfs, M. (2025a). Microsaccades Do Not Give Rise to a Conscious Feeling of Agency for Their Sensorimotor Consequences in Visual Perception. *Journal of Cognition,* 8(1), 51, 10.5334/joc.463. 41142933 PMC12551634

[bib26] Klanke, J.-N., Ohl, S., & Rolfs, M. (2025b). Sensorimotor awareness requires intention: Evidence from minuscule eye movements. *Cognition,* 262, 106176, 10.1016/j.cognition.2025.106176. 40383046

[bib27] Kleiner, M., Brainard, D. H., Pelli, D., Ingling, A., Murray, R., & Broussard, C. (2007). What's new in psychtoolbox-3. *Perception,* 36(14), 1–16.

[bib28] Koerfer, K., Watson, T., & Lappe, M. (2024). Inability to pursue nonrigid motion produces instability of spatial perception. *Science Advances,* 10(45), eadp6204, 10.1126/sciadv.adp6204. 39504371 PMC11540027

[bib29] Kok, E. M., Aizenman, A. M., Võ, M. L.-H., & Wolfe, J. M. (2017). Even if I showed you where you looked, remembering where you just looked is hard. *Journal of Vision,* 17(12), 2, 10.1167/17.12.2. PMC5627674 28973112

[bib30] Kowler, E. (1989). Cognitive expectations, not habits, control anticipatory smooth oculomotor pursuit. *Vision Research,* 29(9), 1049–1057, 10.1016/0042-6989(89)90052-7. 2617852

[bib31] Kowler, E. (2011). Eye movements: The past 25years. *Vision Research,* 51(13), 1457–1483, 10.1016/j.visres.2010.12.014. 21237189 PMC3094591

[bib32] Kowler, E., & Blaser, E. (1995). The accuracy and precision of saccades to small and large targets. *Vision Research,* 35(12), 1741–1754, 10.1016/0042-6989(94)00255-K. 7660582

[bib33] Kowler, E., Kolisetty, L., Aitkin, C., Ross, N., Santos, E., & Shah, R. (2015). Anticipatory smooth eye movements evoked by motor intentions. *Journal of Vision,* 15(12), 1018, 10.1167/15.12.1018.

[bib34] Kowler, E., Martins, A. J., & Pavel, M. (1984). The effect of expectations on slow oculomotor control—IV. Anticipatory smooth eye movements depend on prior target motions. *Vision Research,* 24(3), 197–210, 10.1016/0042-6989(84)90122-6. 6719834

[bib35] Kowler, E., & McKee, S. P. (1987). Sensitivity of smooth eye movement to small differences in target velocity. *Vision Research,* 27(6), 993–1015, 10.1016/0042-6989(87)90014-9. 3660658

[bib36] Kowler, E., Rubinstein, J. F., Santos, E. M., & Wang, J. (2019). Predictive Smooth Pursuit Eye Movements. *Annual Review of Vision Science,* 5(1), 223–246, 10.1146/annurev-vision-091718-014901. 31283450

[bib37] Kowler, E., & Steinman, R. M. (1979). The effect of expectations on slow oculomotor control—I. Periodic target steps. *Vision Research,* 19(6), 619–632, 10.1016/0042-6989(79)90238-4. 547472

[bib38] Kowler, E., & Steinman, R. M. (1981). The effect of expectations on slow oculomotor control—III. Guessing unpredictable target displacements. *Vision Research,* 21(2), 191–203, 10.1016/0042-6989(81)90113-9. 7269296

[bib39] Krauzlis, R. J. (2004). Recasting the Smooth Pursuit Eye Movement System. *Journal of Neurophysiology,* 91(2), 591–603, 10.1152/jn.00801.2003. 14762145

[bib40] Lovejoy, L. P., Fowler, G. A., & Krauzlis, R. J. (2009). Spatial allocation of attention during smooth pursuit eye movements. *Vision Research,* 49(10), 1275–1285, 10.1016/j.visres.2009.01.011. 19533852 PMC2827938

[bib41] Marti, S., Bayet, L., & Dehaene, S. (2015). Subjective report of eye fixations during serial search. *Consciousness and Cognition,* 33, 1–15, 10.1016/j.concog.2014.11.007. 25497406

[bib42] Mcilreavy, L., Freeman, T. C. A., & Erichsen, J. T. (2019). Two-Dimensional Analysis of Smooth Pursuit Eye Movements Reveals Quantitative Deficits in Precision and Accuracy. *Translational Vision Science & Technology,* 8(5), 7, 10.1167/tvst.8.5.7. PMC6753966 31588372

[bib43] Mokler, A., & Fischer, B. (1999). The recognition and correction of involuntary prosaccades in an antisaccade task. *Experimental Brain Research,* 125(4), 511–516, 10.1007/s002210050709. 10323298

[bib44] Morey, R. D., & Rouder, J. N. (2012). *BayesFactor: Computation of Bayes Factors for Common Designs* [Data set]. The R Foundation. 10.32614/cran.package.bayesfactor.

[bib45] Murphy, B. J., Kowler, E., & Steinman, R. M. (1975). Slow oculomotor control in the presence of moving backgrounds. *Vision Research,* 15(11), 1263–1268, 10.1016/0042-6989(75)90172-8. 1198940

[bib46] Nachmani, O., Coutinho, J., Khan, A. Z., Lefèvre, P., & Blohm, G. (2020). Predicted Position Error Triggers Catch-Up Saccades during Sustained Smooth Pursuit. *Eneuro,* 7(1), ENEURO.0196–18.2019, 10.1523/ENEURO.0196-18.2019. 31862791 PMC6964921

[bib47] Nörenberg, W., Schweitzer, R., & Rolfs, M. (2025). Temporal recalibration to delayed visual consequences of saccades. *Journal of Vision,* 25(13), 4, 10.1167/jov.25.13.4. PMC12603963 41201307

[bib48] Pelli, D. G. (1997). The VideoToolbox software for visual psychophysics: Transforming numbers into movies. *Spatial Vision,* 10(4), 437–442, 10.1163/156856897X00366. 9176953

[bib49] Rashbass, C. (1961). The relationship between saccadic and smooth tracking eye movements. *The Journal of Physiology,* 159(2), 326–338, 10.1113/jphysiol.1961.sp006811. 14490422 PMC1359508

[bib50] Rouder, J. N., Speckman, P. L., Sun, D., Morey, R. D., & Iverson, G. (2009). Bayesian t tests for accepting and rejecting the null hypothesis. *Psychonomic Bulletin & Review,* 16(2), 225–237, 10.3758/pbr.16.2.225. 19293088

[bib51] Santos, E. M., & Kowler, E. (2017). Anticipatory smooth pursuit eye movements evoked by probabilistic cues. *Journal of Vision,* 17(13), 13, 10.1167/17.13.13. 29181503

[bib52] Schröder, R., Keidel, K., Trautner, P., Radbruch, A., & Ettinger, U. (2023). Neural mechanisms of background and velocity effects in smooth pursuit eye movements. *Human Brain Mapping,* 44(3), 1002–1018, 10.1002/hbm.26127. 36331125 PMC9875926

[bib53] Schütz, A. C., Braun, D. I., & Gegenfurtner, K. R. (2009). Improved visual sensitivity during smooth pursuit eye movements: Temporal and spatial characteristics. *Visual Neuroscience,* 26(3), 329–340, 10.1017/S0952523809990083. 19602304

[bib54] Schütz, A. C., Braun, D. I., Kerzel, D., & Gegenfurtner, K. R. (2008). Improved visual sensitivity during smooth pursuit eye movements. *Nature Neuroscience,* 11(10), 1211–1216, 10.1038/nn.2194. 18806785

[bib55] Theeuwes, J., Kramer, A. F., Hahn, S., & Irwin, D. E. (1998). Our Eyes do Not Always Go Where we Want Them to Go: Capture of the Eyes by New Objects. *Psychological Science,* 9(5), 379–385, 10.1111/1467-9280.00071.

[bib56] Van Wermeskerken, M., Litchfield, D., & Van Gog, T. (2018). What Am I Looking at? Interpreting Dynamic and Static Gaze Displays. *Cognitive Science,* 42(1), 220–252, 10.1111/cogs.12484. 28295482 PMC5811818

[bib57] Vencato, V., & Madelain, L. (2020). Perception of saccadic reaction time. *Scientific Reports,* 10(1), 17192, 10.1038/s41598-020-72659-3. 33057041 PMC7560701

[bib58] Võ, M. L.-H., Aizenman, A. M., & Wolfe, J. M. (2016). You think you know where you looked? You better look again. *Journal of Experimental Psychology: Human Perception and Performance,* 42(10), 1477–1481, 10.1037/xhp0000264. 27668308 PMC5079107

[bib59] Wong, A. L., & Shelhamer, M. (2012). Using prediction errors to drive saccade adaptation: The implicit double-step task. *Experimental Brain Research,* 222(1–2), 55–64, 10.1007/s00221-012-3195-4. 22850925 PMC3443328

[bib60] World Medical Association. (2013). World Medical Association Declaration of Helsinki: Ethical principles for medical research involving human subjects. *JAMA,* 310(20), 2191–2194, 10.1001/jama.2013.281053. 24141714

[bib61] Wu, G., Yang, Y., & Zeng, L. (2007). Kinematics, hydrodynamics and energetic advantages of burst-and-coast swimming of koi carps (Cyprinus carpio koi). *Journal of Experimental Biology,* 210(12), 2181–2191, 10.1242/jeb.001842. 17562892

[bib62] Yarrow, K., Haggard, P., Heal, R., Brown, P., & Rothwell, J. C. (2001). Illusory perceptions of space and time preserve cross-saccadic perceptual continuity. *Nature,* 414(6861), 302–305, 10.1038/35104551. 11713528

[bib63] Yarrow, K., Whiteley, L., Haggard, P., & Rothwell, J. C. (2006). Biases in the perceived timing of perisaccadic perceptual and motor events. *Perception & Psychophysics,* 68(7), 1217–1226, 10.3758/BF03193722. 17355044

[bib64] Zingale, C. M., & Kowler, E. (1987). Planning sequences of saccades. *Vision Research,* 27(8), 1327–1341, 10.1016/0042-6989(87)90210-0. 3424681

