# Supplementary material for: In pursuit of saccade awareness: Limited volitional control and minimal conscious access to catch-up saccades during smooth pursuit eye movements
Source: J Vis. 2026 Jun 25;26(6):10. doi: 10.1167/jov.26.6.10 (PMC13313219; doi:10.1167/jov.26.6.10)
Supplement: Supplement 1 [file jovi-26-6-10_s001.docx]

# SUPPLEMENTARY MATERIAL

## S0: Supplementary methods: Predicted outcomes

|  | **Analysis** | **Variable** | **Exp.** | **Predicted outcome** |
| --- | --- | --- | --- | --- |
| Stimulus perception | Visual sensitivity | Saccade generation  (zero vs. one saccade) | Exp. 1-3 | Higher visual d’ in trials with generated or replayed catch-up saccades compared to no-saccade trials. |
|  | Visual sensitivity | Stimulus condition (generated vs. replayed) | Exp. 1-3 | No difference in visual d’ between generated and replayed saccades. |
|  | Visual sensitivity | Session | Exp. 2 | No effect of session on visual d’ |
|  | Visual sensitivity | Saccade type  (intended vs. unintended) | Exp. 3 | No effect of saccade type on visual d’ |
|  | Visual sensitivity | Target velocity | Exp. 1-3 | No effect of target velocity on visual d’. |
|  | Visual sensitivity | Retinal velocity | Exp. 1-3 | Higher d’ for low compared to high retinal velocity trials. |
| Voluntary motor control | Saccade rate | Target velocity | Exp. 1-3 | Higher saccade rates at higher target velocities. |
|  | Saccade rate | Session | Exp. 2 | Decline in saccade rates across sessions, reflecting volitional motor control. |
|  | Saccade rate | Saccade type  (intended vs. unintended) | Exp. 3 | Fewer saccades in unintended compared to intended saccade trials. |
|  | Saccade rate | Stimulus presence | Exp. 1-3 | No specific prediction. |
|  | Saccade latency | Target velocity | Exp. 1-3 | No specific prediction. |
|  | Saccade latency | Session | Exp. 2 | Increase in latency across sessions, reflecting ability to delay saccade initiation. |
|  | Saccade latency | Saccade type  (intended vs. unintended) | Exp. 3 | No specific prediction. |
| Saccade awareness | Saccade sensitivity | Stimulus presence | Exp. 1, 3 | Higher saccade d’ in stimulus present compared to absent trials. |
|  | Saccade sensitivity | Stimulus presence | Exp. 2 | Stimulus effect particularly pronounced, as seeing it implied a 75% probability of saccade generation. |
|  | Saccade sensitivity | Session | Exp. 2 | Increase in saccade d’ across sessions, reflecting improved awareness through training. |
|  | Saccade sensitivity | Saccade type  (intended vs. unintended) | Exp. 3 | Above-zero saccade d’ for intended saccades, exceeding d’ for unintended saccades. |
|  | Saccade sensitivity | Target velocity | Exp. 1-3 | No specific prediction. |

**Table S0. Pre-registered hypotheses and predicted outcomes for Experiments 1–3.**

## S1: Causal assignment from Experiment 1

In **Experiment 1**, at the end of each trial in which observers reported seeing the stimulus, we additionally asked them to provide a certainty rating regarding the causal connection between their eye movement and stimulus perception. Specifically, if observers believed they had generated an eye movement, we asked whether they thought this movement caused the change in stimulus visibility. Conversely, if they believed they had not generated an eye movement, we asked how confident they were that the stimulus was not caused by them. Participants could report on a scale using one of four options: not sure, rather unsure, rather sure, and very sure. We included this question to gain insight into participants’ metacognitive awareness of the relationship between their eye movements and the resulting changes in stimulus visibility. We compared this separately for correct assignments (e.g., when a catch-up saccade was generated, the stimulus was seen, and participants reported making the saccade) and incorrect assignments (e.g., when no eye movement occurred, the stimulus was visible due to a replay, but participants still believed they caused the stimulus perception by generating a saccade).

Participants tended to be rather uncertain about the connection between their eye movements and stimulus visibility: average certainty ratings hovered near zero—the center of the scale and the point of highest uncertainty—regardless of stimulus condition (generated: *mean* = 0.20 ± 0.24; replayed: *mean* = 0.15 ± 0.26) or correctness of the assignment (correct: *mean* = 0.32 ± 0.26; incorrect: *mean* = 0.06 ± 0.24; **Fig. S1**). The two-way repeated-measures ANOVA compared average certainty ratings across assignment correctness (correct vs. incorrect) and stimulus condition (generated vs. replayed). Three participants were excluded from this analysis because reliable certainty ratings could not be calculated across all bins. Results showed that neither factor nor their interaction significantly affected certainty (all *p*s > 0.250, all BF_10_ ≤ 0.70), indicating that participants’ uncertainty remained consistent across conditions.


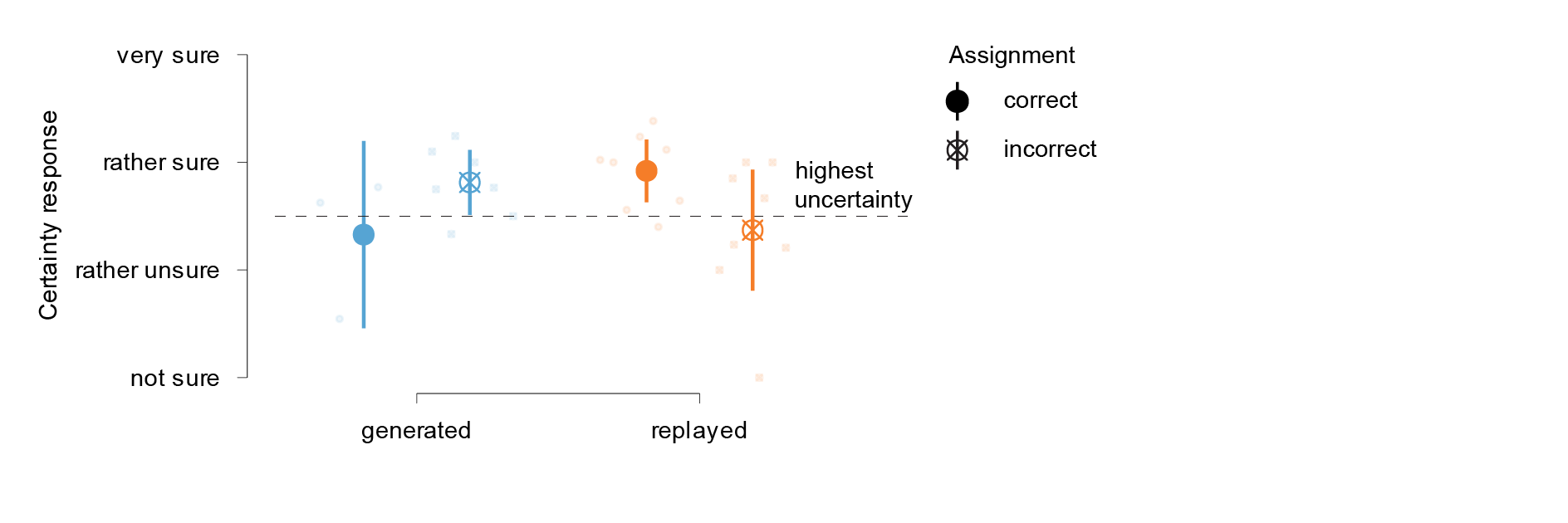


**Fig S1. Low certainty about the causal connection between their eye movements and stimulus visibility—for generated and replayed saccades and irrespective of assignment correctness.** Error bars represent 95% confidence intervals.

Our data indicates that participants’ certainty about the causal connection between their eye movements and stimulus visibility was generally low and unaffected by stimulus type or assignment correctness, suggesting limited metacognitive awareness of the relationship between their actions and perceptual outcomes. Interestingly, participants showed lower certainty and a more heterogeneous response pattern (reflected in larger 95% confidence intervals) when reporting that they had generated an eye movement themselves, regardless of whether this was correct. This again indicates low awareness of saccade generation, suggesting that even when participants believe they caused an eye movement, their confidence in that connection remains weak and variable.

## S2: Observer groups in Experiment 2

We were concerned that training participants over just four sessions to suppress their catch-up saccades might be too short for any potential training effects to emerge. To address this concern and incorporate observer experience into our design, we invited two groups to participate in our second experiment: five naïve observers, who had never taken part in an eye-tracking study, and five expert observers from the lab who had participated in several previous experiments. To assess whether eye movement expertise influenced visual sensitivity, motor control, and awareness of saccades, we repeated all major analyses for **Experiment 2** with the additional factor of observer group.

Focusing on visual sensitivity first, we examined how well participants from each group perceived the stimulus in trials without a catch-up saccade and found sensitivity to be close to zero in both groups (naïve: d’ = 0.10 ± 0.43; expert: d’ = 0.05 ± 0.47; **Fig. S2a**). When turning to trials with a saccade, we found saccade sensitivity to be substantially higher in both observer groups (naïve: d’ = 1.34 ± 1.15; expert: d’ = 1.74 ± 0.70; **Fig. S2a**). A two-way mixed-effects ANOVA confirmed a significant overall increase in sensitivity when a saccade occurred (*F* (1,8) = 25.31, *p* = 0.001), with no significant difference between groups (*F* (1,8) = 0.28, *p* > 0.250) and no interaction (*F* (1,8) = 0.77, *p* > 0.250). A second two-way mixed-effects ANOVA showed that the increase in sensitivity was comparable for generated and replayed eye movements (*F*(1,8) = 0.33, *p* > 0.250), and this pattern held across both observer groups (naïve vs. expert: *F* (1,8) = 0.69, *p >* 0.250; interaction: *F*(1,8) = 0.04, *p* > 0.250).

To determine whether experienced participants had greater volitional motor control over their catch-up saccade generation, we first compared average saccade rates between groups. We found similar rates (naïve: 1.52 ± 0.98 s⁻¹; expert: 1.44 ± 0.67 s⁻¹; **Fig. S2b**), with no statistically significant difference between them (*t* (7.0) = −0.19, *p* > 0.250, BF_10_ = 0.5). To assess potential learning advantages in expert observers, we then calculated saccade rates for each session individually and submitted the data to a two-way mixed-measures ANOVA. This analysis revealed no main effects of session (*F*(3,24) = 0.42, *p* > 0.250) or observer group (*F*(1,8) = 0.03, *p* > 0.250), and no interaction (*F*(3,24) = 1.35, *p* > 0.250), suggesting that saccade suppression performance remained stable over time and did not benefit from prior experience (c.f., **Fig. S2b**). A Bayesian model comparison corroborated these results, with strongest—albeit still low—support for a model including only observer group (BF_10_ = 0.72), while models including session or interactions were substantially less likely (all BF ≤ 0.26).

Lastly, we investigated whether expert observers might be more sensitive to their catch-up saccades or better able to use the saccade-contingent visual feedback to determine whether a saccade had occurred. Irrespective of stimulus presence, saccade sensitivity was similarly low for both naïve (present: d’ = 0.08 ± 0.74; absent: d’ = 0.19 ± 0.73) and expert observers (present: d’ = 0.18 ± 0.81; absent: d’ = 0.03 ± 0.68; **Fig. S2c**). A two-way mixed-measures ANOVA with stimulus presence and observer group as factors confirmed that none of the effects were statistically significant (all *p*s > 0.250). We again corroborated these results using a Bayesian model comparison. The analysis demonstrated that the model including **observer group** was the best-fitting model (BF_10_ = 0.56), though it provided only weak evidence relative to the null. Alternative models including **stimulus presence** (present vs. absent: BF_10_ = 0.40) or interactions (BF_10_ ≤ 0.23) showed somewhat to substantially worse fit, indicating that the addition of these factors did not improve model performance. Overall, the evidence for any effect was weak, with Bayes Factors close to 0.5 reflecting only anecdotal support.

Analyses comparing naïve and expert observers revealed no evidence that prior experience with eye-tracking conferred an advantage in visual sensitivity, motor control, or awareness of catch-up saccades. Both groups exhibited similarly low sensitivity and saccade rates, with no evidence of learning effects across sessions. It stands to reason that eye movement expertise does not enhance awareness or control of catch-up saccades. Additionally, neither awareness nor control likely benefits from long-term training or continued exposure to environments with repeated and tightly controlled eye movement behavior (i.e., piloting or participation of psychophysical experiments).


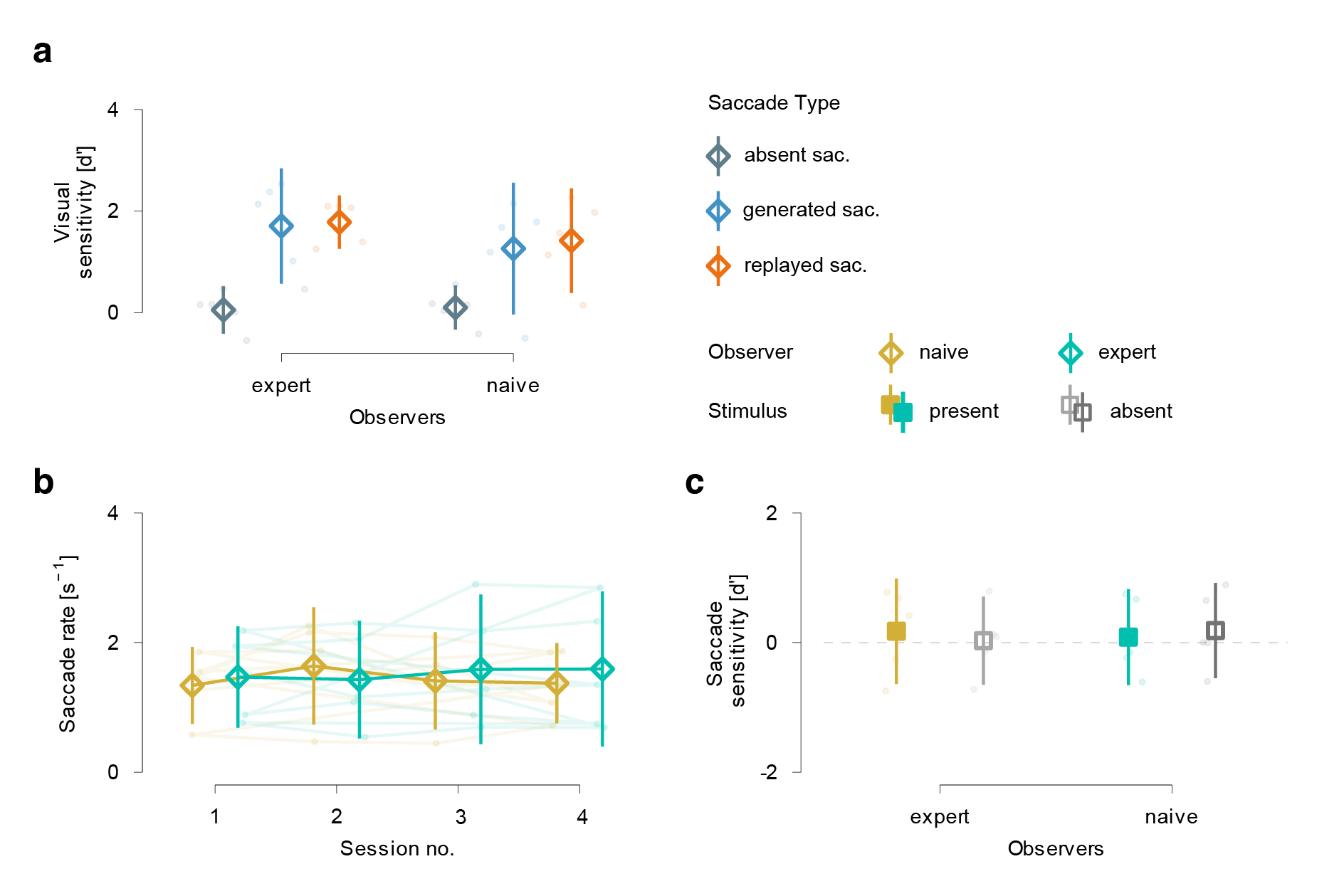


**Fig S2. Pre-training level of the observer has no effect on visual sensitivity, motor control, or saccade sensitivity. a**Visual sensitivity to the stimulus as a function of saccade generation and eye movement condition**.** Data are shown separately for participants with different pre-training levels: naïve and expert. **b**Development of saccade rate (as an index of motor control training) across the four experimental sessions and separately for naïve and expert observers. **c**Saccade sensitivity as a function of stimulus presence and pre-training level. All panels: Error bars represent 95% confidence intervals.

## S3: Saccade parameters in response to target manipulations in Experiment 3

An alternative way to investigate how well observers can control saccade generation during pursuit eye movements is to examine how effectively participants adapted their saccades to the instructed eye movements in **Experiment 3**. Participants were instructed to generate saccades over three distances—0.5, 1.0, or 1.5 dva—either in the direction of the moving target or against it, mimicking forward and backward corrective saccades during pursuit. The go-instruction was presented either early (200–450 ms after trial onset) or late (700–950 ms after trial onset). Late go-cues were included to create conditions in which participants were instructed to saccade but had limited time to comply with the instruction. This allowed us to assess saccade rate as a function of instruction timing, target distance, and saccade direction.

We conducted a three-way rmANOVA with saccade direction (forward vs. backward), target distance, and go-cue timing. The analysis revealed a significant main effect of saccade direction (forward vs. backward: F (1,9) = 5.56, p = 0.043) indicating that participants made slightly but significantly more saccades when instructed to saccade forward (**Fig. S3a**). There was also a significant main effect of target distance (0.5, 1.0, 1.5 dva: F (2,18) = 9.10, p = 0.002), showing that saccade rate increased with increasing saccade amplitude. The main effect of go-cue timing was significant as well (early vs. late: F (1,9) = 16.08, p = 0.003), reflecting a higher saccade rate when the instruction was given early compared to late. Finally, we observed a significant interaction between saccade direction and go-cue timing (F (1,9) = 5.62, p = 0.042), suggesting that the effect of timing differed depending on saccade direction. None of the other interactions were significant (all *p*s ≥ 0.154).

We conducted the same three-way rmANOVA on the first saccade following the go-cue. Here, only two main effects reached significance (**Fig. S3b**): The analysis revealed a significant main effect of target distance (0.5, 1.0, 1.5 dva: *F* (2,18) = 18.67, *p <* 0.001), showing that saccade amplitude increased in line with the instructed target distance. There was also a significant main effect of go-cue time (early vs. late: *F* (2,18) = 6.33, *p =* 0.033), showing that early cues led to slightly larger saccade amplitudes. Unlike in our previous ANOVA for saccade rate, the jump direction of the saccade had no effect (*F* (1,9) = 2.20, *p* = 0.172), nor were there any significant interactions (all *p*s ≥ 0.153).

Together, these analyses show that while saccade rate is influenced by the timing of the go-cue, saccade direction, and target distance, saccade amplitude is primarily driven by saccade direction and target distance but unaffected by go-cue timing. These results provide further evidence that saccade control is possible but primarily shaped by low-level visual factors such as target distance and direction.


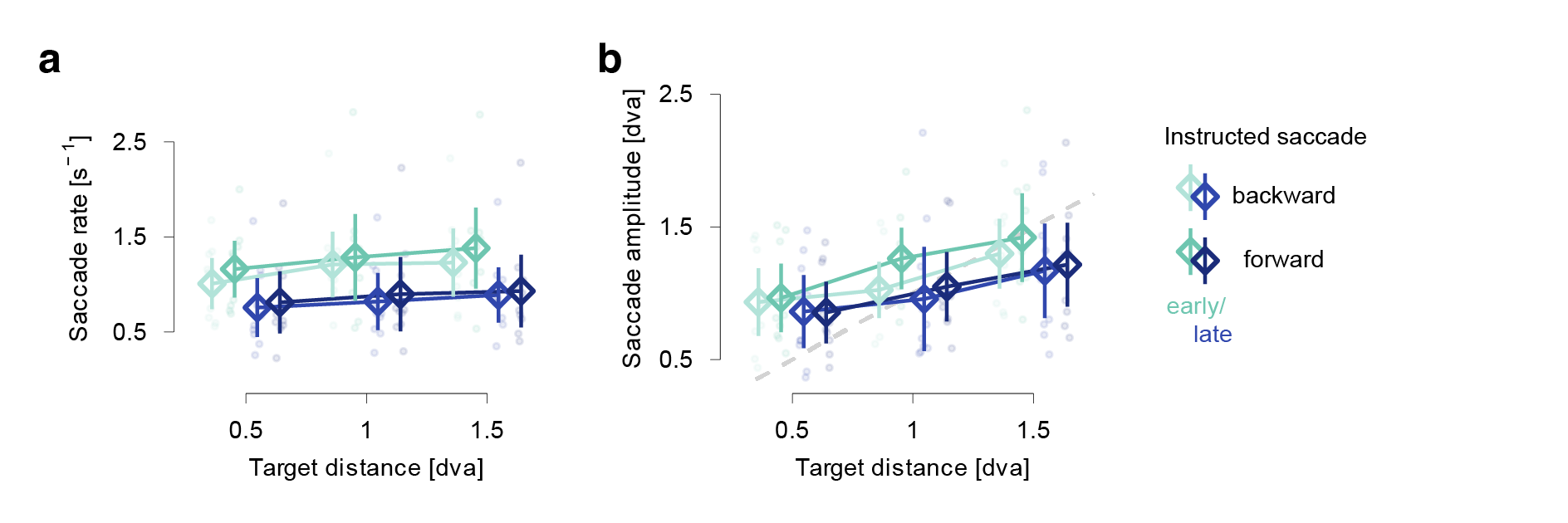


**Fig. S3. Saccade rate and amplitude increase with target distance in instructed catch-up saccade trials. a** Saccade rate as a function of the timing of the go-instruction (early vs. late), the instructed target distance (0.5, 1.0, 1.5 dva), and saccade direction (forward vs. backward) in **Experiment 3**. **b** Saccade amplitude as a function of the same factors. Dashed line marks the origin (x=y). Both panels: Error bars represent 95% confidence intervals.

## S4: A closer look at saccade sensitivity: Hit and false alarm rates across experiments

To gain a more nuanced understanding of saccade awareness, we analyzed hit rates (saccade detection) and false alarm rates (erroneous reports in the absence of a saccade) separately. While a sensitivity measure is better suited to capturing true awareness of catch-up saccades, it does not reveal whether effects were driven by detection accuracy, guessing, or changes in participants’ caution. It might also fail to capture potential effects of the additional factors we investigated—stimulus presence, training, and intention.

To investigate this, we conducted a two-way rmANOVA with the type of response rate and stimulus as factors for **Experiment 1**. The analysis revealed a significant main effect of stimulus presence (present vs absent: F (1,7) = 9.02, p = 0.020), indicating rates were higher when the stimulus was present (hit = 0.18 ± 0.13; fa = 0.15 ± 0.09) compared to stimulus-absent trials (hit = 0.11 ± 0.10; fa = 0.09 ± 0.08). There was no main effect of rate type (hit vs. fa: F (1,7) = 0.59, p > 0.250), suggesting that overall false alarm rates were not significantly different from hit rates. We also found no significant interaction (F (1,7) = 0.60, p > 0.250), indicating that the effect of stimulus presence was similar across both types of responses (**Fig. S4**). These results are broadly supported by our Bayesian model comparison: there was moderate evidence for a model including stimulus presence (BF_10_ = 5.45), anecdotal evidence against a main effect of response rate type (BF_10_ = 0.46), and no clear evidence for an interaction (BF_10_ = 1.13).

To examine what affected response rates in **Experiment 2**, we conducted a three-way rmANOVA with the same two factors as before (stimulus presence and rate type), and session number (to assess training effects) as factors. We found that the main effect of session approached significance (*F* (2.1,19.3) = 3.33, *p* = 0.055 after Greenhouse-Geisser correction for violation of sphericity), as did the main effect of condition (*F* (1,9) = 4.82, *p* = 0.056) and their interaction (*F* (1.9,17.6) = 3.09, *p* = 0.072 after Greenhouse-Geisser correction for violation of sphericity). This pattern is consistent with that observed in the first experiment: in **Experiment 2**, we again found higher response rates during stimulus-present trials (hit = 0.23 ± 0.15; false alarm = 0.20 ± 0.13) than during stimulus-absent trials (hit = 0.09 ± 0.06; false alarm = 0.11 ± 0.07), although these differences did not reach statistical significance. Moreover, hit and false alarm rates did not significantly differ from one another (*F* (1,9) = 0.03, *p* > 0.250), suggesting that stimulus presence influenced overall response tendency rather than selectively affecting detection or guessing (see **Fig. S4**). All remaining interactions remained insignificant (all *p*s ≥ 0.068 after Greenhouse-Geisser correction for violation of sphericity). To complement these effects, we conducted a Bayesian model comparison. The model including **stimulus presence** and participant received the strongest support (BF_10_ = 5.31 × 10^4^), with similar evidence for models that additionally included session (BF_10_ = 1.06 × 10^4^) or response type (BF_10_ = 9.13 × 10^3^). More complex models with interaction terms performed substantially worse, and models omitting **stimulus presence** entirely yielded very low Bayes factors (all BF_10_ ≤ 0.26), indicating that these factors alone poorly accounted for the observed data.

To explore which factors influenced response rates in **Experiment 3**, we conducted a three-way rmANOVA with the same two factors as before (stimulus presence and rate type), and this time included intention (manipulated via pursuit and saccade instruction) to assess the role of volition. The analysis revealed a robust main effect of intention (F (1,9) = 88.16, p < 0.001), indicating that response rates differed markedly depending on whether the eye movement was intentional (hit = 0.82 ± 0.18; FA = 0.80 ± 0.18) or unintentional (hit = 0.11 ± 0.08; FA = 0.08 ± 0.06). The main effect of stimulus presence yet again trended but failed to reach significance (F (1,9) = 4.31, p = 0.068), resembling the data of the first two experiments: participants responded more often on stimulus-present trials (hit = 0.49 ± 0.13; FA = 0.46 ± 0.11) than on stimulus-absent ones (hit = 0.43 ± 0.11; FA = 0.42 ± 0.10). Again, the absence of a main effect of rate type (F (1,9) = 1.49, p > 0.250) suggests that this effect applied similarly to both hits and false alarms, pointing to a general modulation of response likelihood rather than selective changes in detection or guessing. None of the interactions reached significance (all ps > 0.250; c.f. **Fig. S4**). We again conducted a Bayesian model comparison to corroborate the results of the rmANOVA. Our analysis revealed strongest support for models including saccade type and participant (BF_10_ = 1.7 × 10^31^), with models additionally including stimulus presence also receiving substantial support (BF_10_ = 1.1 × 10^31^). Models including only response type (BF = 0.24) showed considerably less support, indicating that saccade type was the primary factor influencing response rates.

Across all three experiments, response rates were consistently influenced by task-related factors rather than by differences in detection. In **Experiment 1**, we found higher response rates when the stimulus was present, regardless of whether the response was a hit or a false alarm, suggesting that visibility alone increased participants’ tendency to report a saccade. **Experiment 2** produced a similar stimulus-driven pattern that varied over time. **Experiment 3**, in turn, revealed a strong effect of intention: participants responded far more often when eye movements were instructed and thus intentional, again with comparable rates for hits and false alarms. Crucially, across all analyses, we found no significant main effects of rate type, indicating that the factors manipulated in the task modulated overall response likelihood rather than selectively affecting perceptual sensitivity. Our data, hence, suggests that awareness of catch-up saccades largely reflect expectations shaped by context and intention, rather than precise introspective access to individual eye movements.


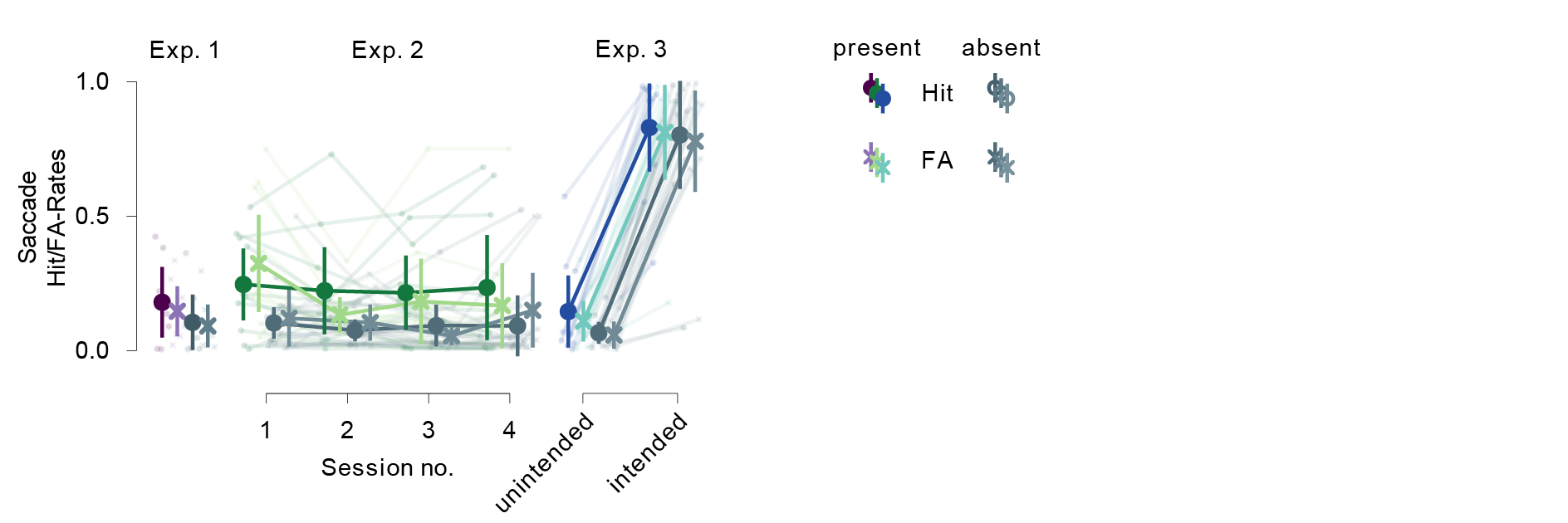


**Fig. S4. Response rates (hit and false alarms) are similarly affected by stimulus presence and intention.** Hit and false alarm rates as a function of stimulus presence, how it develops over time to assess training (**Exp. 2**) and is affected by intention (**Exp. 3**). Error bars represent 95% confidence intervals.

## S5: Smoothed saccade rates.

To quantify saccade dynamics, we calculated smoothed saccade rates for each participant and target velocity condition. Individual saccade times were convolved with a Gaussian kernel (σ = 10 ms) to produce a continuous estimate of saccade probability over time. Rates were then normalized by the number of trials and expressed in events per second (Hz), and finally averaged across participants. Note that saccades occurring during the fixation period were included in this analysis to capture anticipatory responses toward the upcoming moving target.

Across experiments, we observed anticipatory saccade activity during the fixation interval toward the upcoming moving target. This activity was consistent across experiments and target conditions, although its magnitude varied, being strongest in **Experiment 3** (see **Fig. S5**). Anticipatory activity peaked approximately 250 ms before the start of the pursuit interval. We additionally observed early catch-up saccade activity following pursuit onset. Unlike anticipatory responses, these catch-up saccades were modulated by target velocity, with higher rates for faster moving targets. Peak activity occurred roughly 200–300 ms after pursuit onset. This velocity-dependent modulation persisted throughout the trial, most prominently in **Experiment 2**.

Overall, this shows that our paradigm preserved natural catch-up saccade dynamics while also eliciting anticipatory activity, demonstrating that participants engaged both predictive and reactive oculomotor mechanisms, though the strength of these effects varied across experiments.

**Fig. S5. Smoothed saccade rates over time. Smoothed Gaussian saccade rates were first calculated separately for each participant and target velocity condition and then averaged across participants. Error bars represent 95% confidence intervals. Dots on the bands in the top panel represent individual catch-up saccades. Note: Data from the fixation interval are included to illustrate reflexive saccades toward the moving target; these saccades were disregarded in all other analyses (the beginning of the pursuit interval is at 0 ms).**

## S6: Pursuit gain.

To examine how pursuit gain was affected across experiments and manipulations, we computed the unitless velocity ratio between the eye and the target during smooth pursuit, defined as the instantaneous eye velocity divided by the target velocity (values near 1 indicate close matching of target motion). Catch-up saccades were excluded so that gain reflected only the smooth component of the pursuit response. For each participant, gain was averaged across valid time points and then aggregated for each target-velocity condition (**Exp. 1**: 3, 6, 12 dva/s; **Exp. 2** and **3**: 3, 6, 9 dva/s) and for each experimental manipulation (**Exp. 2**: across sessions; **Exp. 3**: comparing intended vs. unintended catch-up saccades).

Overall, participants tracked the target with good accuracy across experiments (**Exp. 1**: mean = 0.85   ± 0.07; **Exp. 2**: mean = 0.84  ±  0.07; **Exp. 3**: mean = 0.89   ±  0.04), with insignificant differences between experiments (*F*(2,25) = 1.01, *p* = 0.380; **Fig. S6**). Target gains were significantly affected by target velocity in **Experiment 1** (*F*(2,14) = 1.19, *p* < 0.001), with higher gains for slower-moving targets (6 dva/s: 0.87   ±  0.07; 9 dva/s: 0.85   ±  0.07; 12 dva/s: 0.82   ±  0.09). Comparable effects were not observed in **Experiment 2** (*F*(2,16) = 3.08, *p* = 0.074) or **Experiment 3** (*F*(2,18) = 2.56, *p* = 0.105).

In **Experiment 2**, gain performance additionally remained stable over time at the group level (*F*(1,8) = 1. 01, *p* > 0.250), with no interaction between target velocity and training (*F*(6,48) = 1.51, *p* = 0.195). Note, however, that this applies only at the group level: participants from the ‘naïve’ population in **Experiment 2** did show significant improvements over time, most notably between the first (very poor) and later (adequate) sessions (cf. **Fig.S6,** middle panel). Interestingly, in **Experiment 3**, the factor **intention** reached significance (*F*(1,9) = 37.77, *p* < 0.001), with higher gain performance in trials with instructed pursuit (0.90   ±0.05) compared to instructed saccade trials (0.88   ±0.04). This effect occurred in the absence of an interaction between target velocity and intention (*F*(2,18) = 0.75, *p* > 0.250), suggesting that providing instructions in **Experiment 3** did not just allow participants to proactively adjust saccade generation, but also enabled them to modulate pursuit performance.

An interesting possibility that could reconcile the suggested voluntary modulation of saccade rate with the low sensitivity for the resulting catch-up saccades is that participants reduced saccade rates by achieving “better” pursuit (i.e., higher pursuit gain). Although the overall increase in gain was small—the gain difference between instructed pursuit and saccade trials was on the order of 0.02—it may have mediated the effect of instruction on saccade generation.


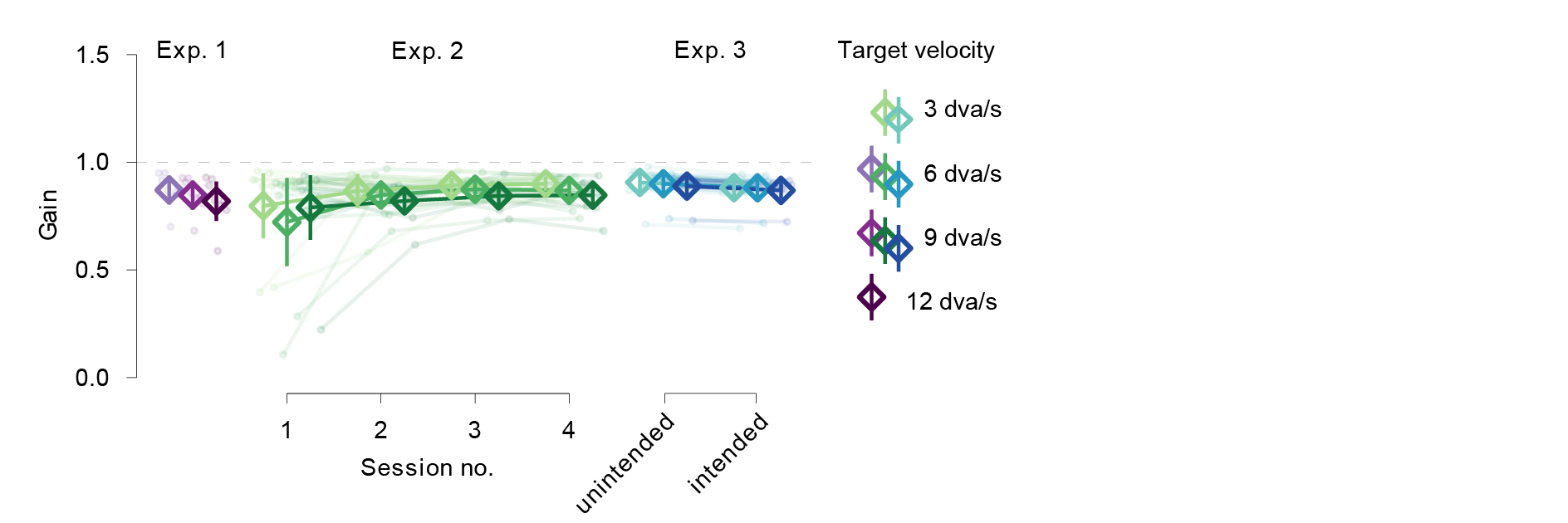


**Fig. S6. Pursuit gain as a function of target velocity, training, and intention.** Group-level pursuit gain across experiments, their development over sessions (i.e., training; **Exp. 2**) and modulation by instructed behavior (**Exp. 3**). Error bars represent 95% confidence intervals.
